# Fasting: From Physiology to Pathology

**DOI:** 10.1002/advs.202204487

**Published:** 2023-02-03

**Authors:** Dongmei Tang, Qiuyan Tang, Wei Huang, Yuwei Zhang, Yan Tian, Xianghui Fu

**Affiliations:** ^1^ Division of Endocrinology and Metabolism, National Clinical Research Center for Geriatrics, State Key Laboratory of Biotherapy, West China Hospital Sichuan University and Collaborative Innovation Center of Biotherapy Chengdu Sichuan 610041 China; ^2^ Neurology Department of Integrated Traditional Chinese and Western Medicine, School of Clinical Medicine Chengdu University of Traditional Chinese Medicine Chengdu Sichuan 610075 China; ^3^ West China Centre of Excellence for Pancreatitis Institute of Integrated Traditional Chinese and Western Medicine West China‐Liverpool Biomedical Research Centre West China Hospital Sichuan University Chengdu Sichuan 610041 China; ^4^ Division of Endocrinology and Metabolism West China Hospital Sichuan University Chengdu Sichuan 610041 China; ^5^ Division of Endocrinology and Metabolism, National Clinical Research Center for Geriatrics, State Key Laboratory of Biotherapy and Cancer Center, West China Hospital Sichuan University and Collaborative Innovation Center of Biotherapy Chengdu Sichuan 610041 China

**Keywords:** aging, diseases, fasting‐mimicking diet, intermittent fasting, time‐restricted feeding

## Abstract

Overnutrition is a risk factor for various human diseases, including neurodegenerative diseases, metabolic disorders, and cancers. Therefore, targeting overnutrition represents a simple but attractive strategy for the treatment of these increasing public health threats. Fasting as a dietary intervention for combating overnutrition has been extensively studied. Fasting has been practiced for millennia, but only recently have its roles in the molecular clock, gut microbiome, and tissue homeostasis and function emerged. Fasting can slow aging in most species and protect against various human diseases, including neurodegenerative diseases, metabolic disorders, and cancers. These centuried and unfading adventures and explorations suggest that fasting has the potential to delay aging and help prevent and treat diseases while minimizing side effects caused by chronic dietary interventions. In this review, recent animal and human studies concerning the role and underlying mechanism of fasting in physiology and pathology are summarized, the therapeutic potential of fasting is highlighted, and the combination of pharmacological intervention and fasting is discussed as a new treatment regimen for human diseases.

## Graphical abstract

Fasting is emerging as a simple but attractive strategy for fighting human diseases, including aging, cancer, neurodegenerative diseases, and metabolic disorders. This review provides an a timely overview of the role and mechanism of fasting in physiology and pathology, highlights the therapeutic potential of fasting, alone and in combination with pharmacological intervention, and discusses the current challenges and prospects.

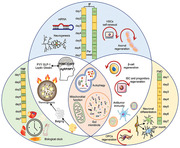

## 1 Introduction

Overnutrition has been recognized as one of the major risk factors for a broad range of human diseases, especially obesity‐related disorders, such as neurodegenerative diseases, metabolic disorders, and cancers. In recent decades, tremendous efforts have been devoted to developing feasible strategies for counteracting overnutrition. To date, two major categories of dietary strategies, namely, calorie restriction (CR) and fasting, have been extensively studied. Accumulating evidence has documented the beneficial effects of both types of interventions on prolonging lifespan and improving healthspan by delaying the onset and slowing the progression of various diseases.^[^ ^1^, ^2^, ^3^, ^4^, ^5^ ^]^

The CR dietary regimen limits the total calorie intake to ≈15–40% relative to ad libitum without malnutrition.^[^ ^1^ ^]^ The effects of CR on delaying aging and increasing healthspan have been studied in a broad spectrum of animals, from monocellular eukaryotes to nonhuman primates.^[^ ^3^, ^6^, ^7^ ^]^ Importantly, increasing evidence from human studies recapitulates numerous key findings from animal experiments.^[^ ^8^, ^9^, ^10^, ^11^ ^]^ For example, it has been shown that 2‐year CR for nonobese individuals has the ability to decrease systemic oxidative damage and promote healthspan.^[^ ^8^, ^12^ ^]^ In addition, CR has profound effects on a series of diseases, such as Alzheimer's disease (AD), Parkinson's disease (PD), multiple sclerosis (MS), cancer, obesity, and diabetes, as well as many other metabolic disorders, as demonstrated by numerous basic and translational research.^[^ ^13^, ^14^, ^15^, ^16^, ^17^, ^18^, ^19^, ^20^ ^]^

Mechanistically, the overall effectiveness of CR seems to be modulated by several factors, including autophagy and inflammation. Autophagy is regulated by nutrient‐sensing signaling pathways,^[^ ^21^ ^]^ particularly the mammalian target of rapamycin (mTOR) and AMP‐activated protein kinase (AMPK) pathways.^[^ ^22^, ^23^ ^]^ CR not only activates AMPK but also inhibits mTOR, leading to an increase in mTORC1, which is responsible for insulin and insulin‐like growth factor (IGF) signals and can suppress autophagy in several ways. For instance, mTORC1 can impair lysosome biogenesis by repressing the transcriptional activity of transcription factor EB (TFEB), thereby reducing the expression of autophagy‐related genes.^[^ ^24^ ^]^ mTORC1 is also able to directly suppress unc‐51 like autophagy activating kinase 1 (ULK1), a component of the autophagy‐related (ATG) complex, which in turn inhibits autophagy.^[^ ^25^ ^]^ Additionally, activation of AMPK, due to an increase in the adenosine triphosphate/adenosine monophosphate (ATP/AMP) ratio, can inhibit the mTOR complex and phosphorylate ULK, thus promoting the initiation of autophagy.^[^ ^26^ ^]^ On the other hand, CR may modulate the immune system in many ways. For instance, CR can regulate the expression of sirtuins (SIRTs), especially activating SIRT1 in the liver, muscle, and brown adipose tissue.^[^ ^27^, ^28^ ^]^ SIRT1 may enhance macrophage function and affect the activation, proliferation, and differentiation of T cells, resulting in a reduced immune response.^[^ ^27^ ^]^ The biological and pathological roles of CR have been extensively discussed elsewhere, and recent excellent reviews are recommended for detailed information.^[^ ^29^, ^30^, ^31^ ^]^

Distinct from CR, fasting refers to the alteration of meal frequency by controlling the time of feeding and prolonging the fast time to hours or even days.^[^ ^1^ ^]^ Recently, fasting as an unconventional approach has attracted considerable critical attention to potentially improve metabolic health. Fasting elicits a series of adaptive changes, such as reducing basal metabolic rates, inducing lipolysis and ketogenesis, modulating hormone concentrations, and decreasing oxidative stress and inflammation.^[^ ^32^, ^33^, ^34^, ^35^ ^]^ Correspondingly, numerous preclinical and clinical studies have demonstrated the benefits of fasting in preventing and/or resisting multiple diseases.^[^ ^36^, ^37^, ^38^, ^39^ ^]^ Here, we aim to provide a timely and comprehensive view of the multifaceted role of fasting in both physiological and pathological states. We thus summarize the cellular and systemic responses of fasting, outline recent preclinical and clinical applications of fasting regimens in human diseases, and discuss the current challenges and future prospects.

## 2 Fasting in Physiology

**Figure 1:**
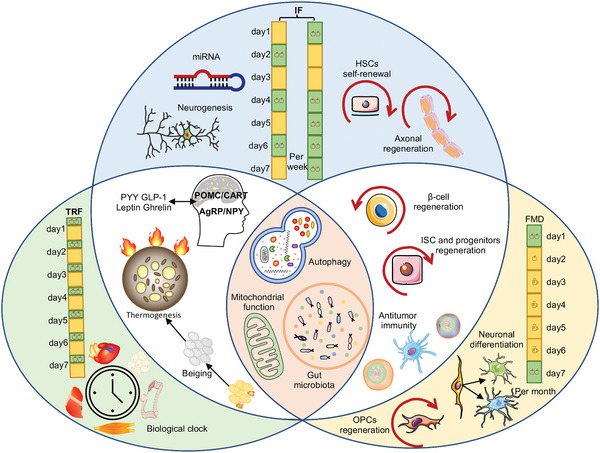
Three fasting schemes and their mechanisms. TRF refers to an eating pattern in which the food intake is restricted to a time window of 4–12 h every day. The effects of TRF are mainly controlled by peripheral tissue circadian clocks. IF is characterized by periods of little or no energy intake, which can last a day or more. ADF and the “5:2” diet are two widely used paradigms. Different from the other two regimens of fasting, IF can interact with ncRNA and thus regulate hepatic glucose and lipid metabolism. HSCs self‐renewal and axonal regeneration are also only achieved by IF regimens. FMD is characterized by decreasing caloric intake for only 5 days each month. The benefit and mechanism of FMD link with the regeneration and differentiation of multiple tissues and cells, including *β*‐cell, ISCs, OPCs, and neurons. Both TRF and IF can affect appetite and accelerate fat mobilization. Consistent with FMD, IF also facilitates *β*‐cell and ISCs regeneration, as well as enhances host antitumor immune response. In spite of the differences in core molecular machinery and signaling regulation, studies in animals and humans showed that three fasting regimens can affect autophagy, gut microbiota, and mitochondria.

Currently, there are three representative patterns of fasting regimens, namely, time‐restricted feeding (TRF), intermittent fasting (IF), and fasting‐mimicking diet (FMD).^[^ ^1^ ^]^ TRF is a daily fasting pattern in which all nutrient intake occurs within a few hours (typically 4–12 h) every day without calorie reduction.^[^ ^1^ ^]^ IF refers to alternate periods of eating and extended fasting.^[^ ^40^ ^]^ FMD is a low‐calorie plant‐based diet program that provides sufficient micronutrient nourishment, including vitamins and minerals.^[^ ^41^ ^]^ All three fasting regimens have been shown to have various positive health impacts, although they may preferentially exert their effects on distinct physiological aspects via similar or different mechanisms (**Figure** **1**).^[^ ^40^, ^42^, ^43^ ^]^ Next, we will summarize the physiological roles and underlying mechanism of the three fasting modes.

### 2.1 Time‐Restricted Feeding

TRF is somewhat inspired by certain religious rituals.^[^ ^44^, ^45^ ^]^ For instance, Muslims fast from dawn until dusk during the month of Ramadan, while Christians, Jews, Buddhists and Hindus traditionally fast on designated days of the week or calendar year.^[^ ^46^, ^47^ ^]^ Currently, TRF refers to food consumption that is restricted to certain hours of the day without calorie restriction, usually spanning 4 to 12 h (Figure 1).^[^ ^1^, ^40^, ^48^ ^]^

**Figure 2:**
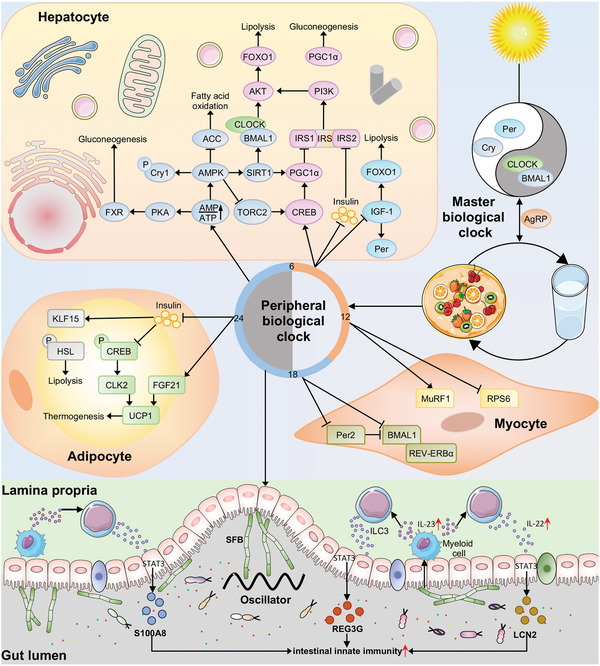
TRF adjusts the peripheral circadian clock. The circadian clock system in peripheral tissues of mammals is entrained by light‐dark and fasting‐feeding cycles. TRF resets the circadian clocks in peripheral tissues, including the liver, muscle, adipose tissues, as well as gut microbiota. In the liver, TRF regulates glucose and lipid metabolism by activating temporal genes (e.g., AMPK and CREB) and decreasing insulin and IGF‐1 levels. TRF also promotes thermogenesis and lipolysis of adipocytes mostly by inhibiting insulin secretion and regulating rhythmic expression of key genes. In the muscle, TRF mainly influences the expression of Per and Baml1, which are important regulators of the circadian clock. In addition, TRF induces the diurnal oscillations of gut bacteria, which can affect various physiological processes in host cells. For example, rhythmic SFB attachment drives the rhythms of antimicrobial proteins, including S100A8, REG3G, and LNC2, which contribute to the intestinal innate immunity.

The vast majority of studies have suggested that most of the benefits of TRF, including improved insulin sensitivity, might depend on the perfect alignment of the fasting‐feeding cycle with the circadian system (**Figure** **2**). The circadian system is a distinctive feature of nearly all living organisms and regulates a wide diversity of physiological processes, including blood pressure, release of endocrine hormones, body temperature, and metabolic activity.^[^ ^49^, ^50^ ^]^ Circadian rhythms, which are monitored by molecular clocks, are highly conserved internal timing mechanisms enabling cells, organs, and animals to anticipate and thereby adapt to the daily changes in their environment, such as activity‐rest and energy intake.^[^ ^50^, ^51^ ^]^ Basically, circadian clocks consist of the master circadian pacemaker located in the suprachiasmatic nucleus (SCN) of the hypothalamus and the secondary clocks in the peripheral organs and tissues, including liver, heart, muscle, and adipose tissue.^[^ ^52^ ^]^ The SCN clock is entrained by changes in the light/dark cycle and synchronizes peripheral circadian rhythms.^[^ ^51^, ^53^ ^]^ At the molecular level, the circadian clock system is controlled by four key circadian genes, namely, brain and muscle Arnt‐like protein 1 (Bmal1), circadian locomotor output cycles kaput (Clock), cryptochrome (Cry), and period (Per).^[^ ^54^ ^]^ Interestingly, these four genes form a transcription‐translation feedback loop, in which Bmal1 and Clock promote the transcription of Per and Cry, while the Per/Cry complex provides negative feedback on the activity of Bmal1 and Clock.^[^ ^55^, ^56^ ^]^

TRF entrains the peripheral circadian clock, especially the liver clock (Figure 2). The feeding schedule has a prominent effect on the expression of rhythm‐related genes in peripheral organs and tissues, including liver, adipose tissue, and muscle. Specifically, restricted feeding plays an essential role in modulating the peripheral circadian clock in the liver.^[^ ^57^, ^58^, ^59^ ^]^ The hepatic biological clock can be set or reset by mealtime, which is achieved by driving the rhythmic transcription of thousands of genes involved in diverse physiological processes, such as agouti‐related neuropeptide (AgRP) and AMPK.^[^ ^57^, ^60^ ^]^ Accumulating evidence suggests that TRF is particularly compatible with liver rhythms by modulating the expression of multiple rhythmic regulators (Figure 2). TRF can rapidly induce the expression of AgRP by diminishing the expression of small‐conductance calcium‐activated potassium channel 3,^[^ ^61^ ^]^ while AgRP is critical to enhance the expression of hepatic rhythm‐related transcription factors, including Per, Cry, Clock and Bmal1.^[^ ^62^ ^]^ TRF can also activate the fasting‐sensitive protein kinase AMPK by increasing AMP levels.^[^ ^59^ ^]^ AMPK can modulate the circadian clock by regulating key circadian clock regulators. For example, AMPK can phosphorylate CRY1 and facilitate its degradation by enhancing the CRY1‐F‐box and leucine rich repeat protein 3 (FBXL3) interaction.^[^ ^63^ ^]^ In line with the key role of the AMPK pathway in cellular metabolism, TRF has been shown to maintain metabolic homeostasis by restoring the expression rhythm of peripheral clock genes in SCN clock‐deficient mice.^[^ ^64^ ^]^ Hatori et al. reported that an 18‐week TRF regimen in mice led to a moderate increase in hepatic AMP levels, indicative of increased AMPK activity, along with improved overt rhythms.^[^ ^59^ ^]^ Totally, TRF can reset circadian clock by influencing AgRP‐controlled key circadian genes (including Per, Cry, Clock, and Bmal1) and increasing the ratio of AMP/ATP in liver.

In addition, TRF may regulate circadian rhythms in muscle, white adipose tissue (WAT), gut microbiota, and other tissues (Figure 2).^[^ ^57^, ^58^, ^65^, ^66^, ^67^, ^68^ ^]^ TRF improves the muscle circadian rhythm by modulating the clock master genes, such as BMAL1 and Per, as well as critical clock‐responsible regulators including tripartite motif containing 63 (TRIM63, also known as MuRF1) and ribosomal protein S6 .^[^ ^57^, ^68^, ^69^ ^]^ In line with these effects, TRF plays an important role in maintaining the daily rhythm in muscle mitochondrial respiration, and can reset obesity‐induced disruption of the muscle clock and restore muscle function.^[^ ^66^, ^70^ ^]^ TRF can restore the dysregulated WAT clock caused by constant light exposure, albeit the underlying mechanisms remain unclear.^[^ ^58^ ^]^ Nevertheless, it has been shown that TRF could affect the circadian rhythms of several regulators crucial for adipose function, including fibroblast growth factor 21 (FGF21),^[^ ^71^ ^]^ and hormone‐sensitive lipase (HSL).^[^ ^72^ ^]^ For example, TRF might prevent high dietary fat‐triggered disruption of FGF21 circadian oscillation, resulting in decreased uncoupling protein 1 (UCP1) proteins in WAT and thermogenesis.^[^ ^71^ ^]^ Moreover, TRF may be associated with the intestinal microbiota rhythmicity. TRF can induce the rhythmic attachment in intestinal epithelial cells of segmented filamentous bacteria (SFB). Rhythmic SFB attachment drives diurnal rhythms in the expression and activation of signal transducer and activator of transcription 3 (STAT3), a key regulator for immune response, which modulates the rhythms of antimicrobial proteins, including regenerating islet‐derived protein 3*γ* (REG3G), lipocalin‐2 (LCN2) and S100A8, and thus stimulates intestinal innate immunity.^[^ ^73^ ^]^ Other studies also showed that TRF can adjust the gut microbiota,^[^ ^74^, ^75^, ^76^ ^]^ however the relationship with circadian rhythms is currently unknown. Although increasing evidence suggests that the benefits of TRF are dependent on a functional circadian clock, a previous study showed that TRF can still alleviate metabolic stress and prevent metabolic disorders in mice lacking a circadian clock.^[^ ^77^ ^]^ These results indicate that the contribution of the circadian clock to TRF effects may be context‐dependent, and future investigations are needed to further clarify this hypothesis. For example, it is of interest to examine circadian gene expression in various models with different genetic backgrounds or fasting durations.

In agreement with its role in the circadian clock, TRF can modulate the expression of a series of temporal genes, especially cAMP‐response element binding protein (CREB), and play a role in energy homeostasis (Figure 2). TRF can induce the rhythmicity of hepatic CREB activity. Per2, a circadian master gene responsive to TRF, not only facilitates the interaction between CREB and its coregulator CREB‐regulated transcription coactivator 1 (CRTC1) but also promotes the recruitment of the histone acetyltransferase CREB binding protein (CBP) to CREB.^[^ ^78^ ^]^ In turn, the CREB‐CRTC1‐CBP complex is capable of stimulating the transcription of genes containing a cAMP response element, such as Per1 and Per2.^[^ ^78^, ^79^ ^]^ Accordingly, the circadian clock has been reported to modulate gluconeogenesis through the CRY‐cAMP‐CREB axis.^[^ ^80^, ^81^ ^]^ The phosphorylation and activation of CREB drive the transcription of crucial gluconeogenic genes, including phosphoenolpyruvate carboxykinase 1 and glucose‐6‐phosphatase. CREB is also a critical activator of peroxisome proliferator‐activated receptor *γ* (PPAR*γ*) coactivator 1*α* (PGC1*α*).^[^ ^82^ ^]^ The CREB/PGC1*α* axis may activate multiple gluconeogenic genes during fasting to enhance hepatic gluconeogenesis, lipid catabolism, and detoxification of reactive oxygen species produced by mitochondria.^[^ ^82^ ^]^ In addition, TRF activates the protein kinase A (PKA) signaling pathway by inducing farnesoid X receptor (FXR) and CREB and thus enhances fasting hepatic gluconeogenesis (Figure 2).^[^ ^83^ ^]^

In addition to CREB, fasting regulates a switch in the expression of other temporal genes by dedicating fasting‐sensitive factors, such as IGF‐1, extracellular regulated protein kinases (ERKs), forkhead box transcription factor class O (FOXO), and PPAR, which are involved in the metabolism of glucose and lipids (Figure 2).^[^ ^57^, ^60^, ^68^, ^82^, ^84^, ^85^, ^86^ ^]^ The endocrine and nutrient‐sensing insulin/IGF‐1/FOXO pathway has been highly conserved during evolution, and IGF‐1 is an essential autocrine and paracrine factor for cell survival, proliferation, growth, and differentiation.^[^ ^87^ ^]^ However, high levels of IGF‐1 can promote tumorigenesis, as well as the development of aging and metabolic diseases.^[^ ^88^, ^89^ ^]^ Circulation of IGF‐1, primarily originating from the liver, in the blood is tightly regulated by the hepatic circadian clock.^[^ ^90^ ^]^ TRF decreases the expression of IGF‐1 and inhibits insulin secretion during fasting, and then restores IGF‐1 and insulin levels during refeeding. This virtuous cycle modulates the resetting of the circadian clock.^[^ ^60^ ^]^ In addition, TRF can regulate the expression of kruppel‐like factor 15 (KLF15) to maintain energy homeostasis by accelerating lipolysis during fasting and promoting lipid synthesis in the course of refeeding.^[^ ^91^ ^]^ These findings suggest that TRF could improve systemic glucose and lipid metabolism via aligning circadian clock and driving rhythmic expression of a series of genes.

Apart from circadian rhythms, TRF has been found to play an important role in regulating appetite (Figure 1). The control center for appetite is located in the hypothalamus, which is controlled by antiappetite neurons (pro‐opiomelanocortin (POMC) and cocaine–amphetamine‐regulated transcript (CART) neurons) and appetite‐promoting neurons (neuropeptide Y (NPY) and AgRP neurons).^[^ ^92^, ^93^, ^94^ ^]^ TRF influences appetite by promoting NPY/AgRP neurons to release AgRP.^[^ ^95^ ^]^ Additionally, TRF can regulate appetite by hormones and peptides, such as leptin, insulin, and ghrelin.^[^ ^96^ ^]^ Leptin and insulin inhibit food intake by activating POMC/CART neurons and inhibiting NPY/AgRP neurons in the arcuate nucleus.^[^ ^97^, ^98^ ^]^ As opposed to insulin and leptin, ghrelin is an orexigenic hormone that can suppress POMC/CART neurons and stimulate NPY/AgRP neurons.^[^ ^98^ ^]^ Rodent studies suggest an inhibitory effect of TRF on appetite and body weight, while human clinical and translational studies either support or oppose these effects.^[^ ^85^, ^99^, ^100^, ^101^ ^]^ These discrepancies may be due to the differences in TRF modes, study design, or cohort population. For instance, compared to mid‐day or late TRF (12‐h feeding period) in humans, an early TRF (6‐h feeding period during daytime) decreased insulin and leptin and reduced appetite in the evening.^[^ ^85^, ^102^, ^103^ ^]^ In addition, TRF can decrease endogenous ghrelin levels and reduce appetite in adults who are overweight or healthy.^[^ ^99^, ^104^ ^]^ These findings collectively suggest that TRF may affect appetite by modulating appetite‐related neuronal signals and hormones, however, it is of significance for future studies to clarify its different effects and underlying mechanisms on humans and rodents.

Altogether, these recent findings indicate that TRF could be a promising treatment for improving health. Despite these tremendous advances, the molecular mechanisms underlying the physiological and pathological effects of TRF are not yet completely understood. In particular, current studies mainly focus on the role of TRF in peripheral circadian clocks,^[^ ^60^, ^77^ ^]^ however, knowledge of the effects of the direct association between TRF and the master circadian clock is quite limited and awaits further investigation. Furthermore, particular attention should be given to emerging TRF‐related adverse effects or uncertain results. For example, TRF during childhood may have persistent effects on gut microbiota and cause irreversible metabolic disorders.^[^ ^105^, ^106^ ^]^

### 2.2 Intermittent Fasting

IF, periods of voluntary abstinence from food and drink, is an ancient practice with a variety of different formats for populations globally.^[^ ^107^ ^]^ In recent years, IF has become popular due to its safety and effectiveness, based on a large number of studies.^[^ ^108^, ^109^, ^110^ ^]^ IF is characterized by recurring periods with little or no energy intake that range from one to several days.^[^ ^1^ ^]^ Although various IF regimens have been proposed, the two most popular and well‐studied approaches are alternate day fasting (ADF) and 2 days of fasting or severe restriction (75–90% of energy needs, the 5:2 IF) followed by a 5 consecutive days ad libitum eating period per week (Figure 1).^[^ ^111^, ^112^, ^113^, ^114^ ^]^

**Figure 3:**
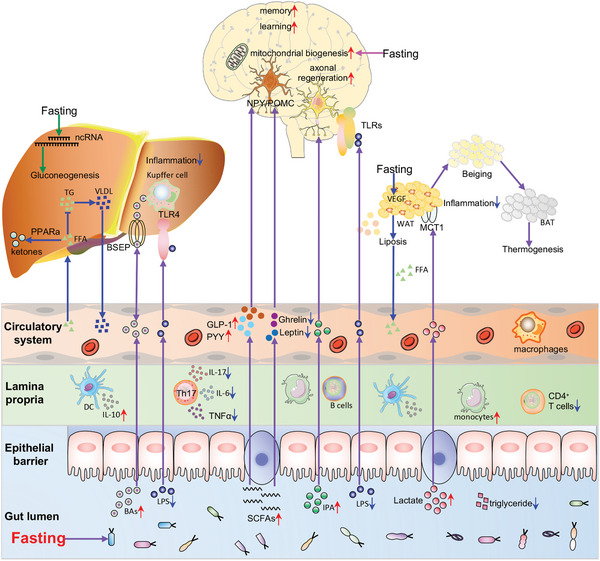
Fasting can mediate inter‐organ communication. On the one hand, fasting affects gut microbiota and its metabolites, thereby influencing immune modulatory effects. For instance, SCFAs can influence the differentiation, recruitment, and activation of various immune cells, including DCs, macrophages, monocytes, and T cells. On the other hand, fasting can reshape the gut microbiota and affect the production of gut microbiome‐generated metabolites, such as BAs, SCFAs, IPAs, and LPS, which in turn influence host physiology. Fasting‐induced accumulation of BAs is reabsorbed into the enterohepatic circulation by the ileal bile acid transporter and is transported to the liver via the portal blood circulation, thereby influencing liver physiology. Similarly, fasting‐mediated hormones and metabolites can influence several specific brain regions and neurons via the circulation. SCFAs simulate the secretion of gut hormones GLP‐1 and PYY from enteroendocrine L cells and thus affect appetite and some cognitive functions, including memory and learning. IPA production by *Clostridium sporogenes* significantly enhances axonal regeneration. LPS can directly act on TLRs in the brain and modulate neuroinflammatory processes, thereby modifying brain function. Gut microbiome‐generated metabolites also mediate gut‐adipose tissue communication. For example, lactate was transported into cells through MCT1 in white adipocytes to promote mobilization of adipose tissue. In addition to gut microbiota, fasting can directly regulate brain function, liver metabolism, and fat mobilization in white adipocytes. FFAs generated by adipolysis are circulated to the liver for the generation of ketone bodies.

Numerous studies have demonstrated that IF can shape the gut microbiome and in turn influence host physiology, particularly immunity and metabolism (**Figure** **3**). It is widely accepted that intestinal microflora plays an important role in human health and disease.^[^ ^115^, ^116^ ^]^ As a critical component of digestion, the gut microbiome breaks down the nutritional composition and alleviates metabolic stress.^[^ ^117^, ^118^ ^]^ Moreover, it may regulate host metabolism, immunity, and even behavior through its metabolites.^[^ ^118^ ^]^ IF can directly result in rapid shifts in the composition and function of the gut microbiome by modulating the abundance of specific species and their individual or collective functions.^[^ ^119^, ^120^ ^]^ Rodent studies have shown that IF could increase the relative abundance of probiotics within the gut, such as the *Lactobacillaceae*, *Bacteroidaceae*, and *Firmicutes microbial* families,^[^ ^120^, ^121^, ^122^ ^]^ which might have a role in reducing chronic intestinal inflammation or increasing utilization of dietary energy and nutrients.^[^ ^120^, ^123^, ^124^ ^]^ Similarly, IF may increase the levels of Bacteroidaceae and Firmicutes.^[^ ^125^, ^126^, ^127^ ^]^ *Bacteroidetes* and *Firmicutes* comprise the majority of bacteria in the mammalian intestine and dominate gut microbiota biodiversity.^[^ ^128^ ^]^ They ferment dietary fibers and produce short chain fatty acids (SCFAs).^[^ ^129^ ^]^ In line with these results, IF lasting for 15 cycles enhanced the production of SCFAs, including acetate, propionate, and butyrate.^[^ ^122^, ^130^, ^131^ ^]^ SCFAs can act on G‐protein coupled receptors in enteroendocrine cells and influence immune modulatory effects.^[^ ^132^ ^]^ Functionally, SCFAs can influence the differentiation, recruitment, and activation of various immune cells, including neutrophils, dendritic cells (DCs), macrophages, monocytes, and T cells (Figure 3).^[^ ^132^, ^133^, ^134^ ^]^ These functions of SCFAs are in accord with the consequences of IF. For instance, it has been recently shown that IF can enhance gut mucosal immune responses by reducing the number of B and T lymphocytes in Peyer's patches (PPs).^[^ ^135^ ^]^ Studies in both animal models and human subjects have suggested that IF can increase the number of peripheral blood lymphocytes and alleviate inflammation.^[^ ^109^, ^120^, ^136^ ^]^ Altogether, these results demonstrate a function of IF on host immunity through modulating gut microbiota.

Meanwhile, IF may have a role in systemic metabolism and homeostasis, partially due to its effects on the production of gut microbiome‐generated metabolites (Figure 3). These metabolites, such as SCFAs and lipopolysaccharide (LPS), may mediate multiple interorgan interactions, including the gut‐brain, gut‐adipose, and gut‐liver axis (Figure 3). For instance, SCFAs simulate the secretion of gut hormones glucagon‐like peptide‐1 (GLP‐1) and peptide YY (PYY) from enteroendocrine L cells, but inhibit the release of ghrelin and leptin.^[^ ^137^, ^138^ ^]^ These hormones are known to affect appetite, mood, and some cognitive functions, including memory and learning.^[^ ^139^, ^140^ ^]^ For example, GLP‐1 can enhance glucose‐dependent insulin secretion and inhibit glucagon release.^[^ ^141^ ^]^ Of note, IF can reduce the levels of circulating LPS by increasing the abundance of *Bacteroides spp*. to suppress LPS biosynthesis and enhancing the gut barrier to decrease LPS release.^[^ ^121^, ^142^ ^]^ LPS can act on toll‐like receptors (TLRs) in the brain and participate in some neuroinflammatory processes;^[^ ^143^ ^]^ thus, IF has a role in preserving brain function.^[^ ^121^, ^142^ ^]^ In addition, it has been shown that 3 or 15 cycles of IF in mice can increase the circulating levels of lactate, a metabolite derived from intestinal flora.^[^ ^122^ ^]^ Serum lactate can be transported across the plasma membrane of adipocytes through monocarboxylate transporter 1 (MCT1) and thus induce inguinal WAT beiging.^[^ ^122^ ^]^ These results collectively suggest that IF may modulate interorgan communication by regulating intestinal flora and their metabolites.

In addition to the gut microbiome and its metabolites, PPAR*α* may contribute to the effect of IF on systemic metabolism (Figure 3). PPAR*α* is a transcription factor that can be activated by fatty acids and numerous other lipid species.^[^ ^144^ ^]^ Intriguingly, PPAR*α* is an important nexus for the metabolic network driven by multiple nutrient sensors, including AMPK, mTOR, PGC1*α*, and FGF21, and plays an essential role in whole‐body metabolism.^[^ ^144^, ^145^ ^]^ In particular, PPAR*α* is highly expressed in the liver and functions as a major regulator of hepatic fatty acid oxidation (FAO) and ketogenesis.^[^ ^146^ ^]^ PPAR*α* deficiency in mice impairs acute fasting‐induced increases in FAO gene expression and plasma ketone body levels.^[^ ^147^ ^]^ Although the significance of PPAR*α* in systemic metabolism under both physiological and pathological conditions is well known, its implication in fasting regimens is just emerging. Fasting can induce PPAR*α*, but suppress mTORC1 activity, which may contribute to some consequences of fasting, such as increased rate of hepatic FAO, elevated levels of serum ketone bodies, and reduced levels of plasma free fatty acids and lipid accumulation.^[^ ^5^, ^120^, ^148^, ^149^, ^150^, ^151^ ^]^ Interestingly, Li et al. showed that fasting every other day protected mice against fasting‐induced steatosis independent of PPAR*α*.^[^ ^152^ ^]^ This inconsistency is probably due to different experimental models or periods of IF cycles. It would be interesting to clarify the role and mechanism of PPAR*α* in IF, as well as other fast regimens, in the future.

Moreover, IF may influence many other organs and tissues, such as the digestive, reproductive, immune, and circulatory system, to improve health. For instance, IF is able to restore autophagic flux and improve glucose tolerance by increasing glucose‐stimulated insulin secretion in pancreatic islets, thereby preventing hyperglycemia, insulin resistance, and progressive *β*‐cell failure.^[^ ^153^, ^154^ ^]^ Notably, starvation can increase reproductive longevity at least 15‐fold and extend total adult lifespan at least 3‐fold in C. elegans,^[^ ^155^ ^]^ indicative of a speculative role of IF in the reproduction system. As to the immune system, IF can enhance the mucosal immune response by reducing the number of lymphocytes in PPs and activate antimicrobial defense in intestinal epithelial cells through the hes family bHLH transcription factor 1‐atonal bHLH transcription factor 1‐matrix metallopeptidase 7 signaling pathway.^[^ ^135^, ^156^ ^]^ More recently, a randomized controlled clinical trial showed that IF led to beneficial changes in the cardiovascular system, circulating metabolites, systemic metabolism, and body composition,^[^ ^109^ ^]^ highlighting a complex and versatile effect of IF on multiple organs. In addition, IF may play a role in stem cell biology and thus modulate tissue differentiation and homeostasis (Figure 1). It can improve the self‐renewal capacity of hematopoietic stem cells (HSCs) and promote HSC regeneration and rejuvenation.^[^ ^157^ ^]^ Similarly, 24‐h fasting is beneficial to the function of intestinal stem cells (ISCs) in both young and old mice, including promoting FAO and ISC regeneration.^[^ ^158^ ^]^ It is of great interest for future studies to dissect the contribution of different target organs/systems to the beneficial phenotype of IF.

Despite a remarkable increase in the understanding of the outcomes of IF on health, an understanding of the underlying molecular mechanisms has lagged behind, likely due to the multifaceted roles of IF on various tissues. Interestingly, accumulating evidence suggests that an intricate interaction between IF and noncoding RNAs (ncRNAs) may mediate the functions of IF (Figure 3). ncRNAs, particularly microRNAs (miRNAs) and long noncoding RNAs (lncRNAs), play diverse roles in almost all biological, physiological and pathological processes.^[^ ^159^, ^160^, ^161^, ^162^ ^]^ IF can affect the levels of numerous miRNAs by modulating the expression of miRNA‐induced silencing complex (miRISC) components, including Argonaute and GW182, and the miRNA‐processing enzyme DROSHA.^[^ ^163^ ^]^ Moreover, IF may regulate certain miRNAs under specific conditions. For instance, IF is able to increase the expression of some hepatic miRNAs, including miR‐15a, miR‐17, and miR‐29c, and then participates in several metabolic and mitochondrial pathways.^[^ ^164^ ^]^ IF can inhibit miR‐29a expression and thus suppress lipoprotein lipase, but induce the expression of miR‐130a that directly targets histone deacetylase 3 (HADC3), a marker of AD, resulting in improved brain function and reduced risk for AD.^[^ ^165^, ^166^ ^]^ In addition to miRNAs, lncRNAs might contribute to the effects of IF on glucose metabolism and metabolic stress. For instance, the lncRNA liver glucokinase repressor (lncLGR) induced by fasting can drive the recruitment of hnRNPL to the glucokinase (GCK) promoter, leading to reduced GCK transcription and glycogen storage.^[^ ^167^ ^]^ Gm15441, an inducible liver‐specific lncRNA responsive to PPAR*α* activation, can suppress its antisense transcript encoding thioredoxin interacting protein and subsequently prevent hepatic inflammation during acute fasting.^[^ ^168^ ^]^ Given the tremendous number of ncRNA genes and their broad role in both physiology and pathology,^[^ ^169^, ^170^, ^171^ ^]^ a deep understanding of the interactions between ncRNAs and IF is highly anticipated.

Taken together, these studies demonstrate that IF may benefit health by improving the function and homeostasis of multiple organs and tissues, including intestinal microflora, peripheral tissues (the liver, adipose tissue, and pancreas), the immune system, and stem cells, and indicate ncRNAs as important mediators of IF functions (Figures 1 and 3). However, some physiological alternations for IF remain unclear. For instance, unlike TRF or ADF, the 5:2 IF regimen is an unbalanced alternating diet, which may have potential effects on circadian rhythm and the endocrine systems.^[^ ^45^ ^]^ However, at present, studies regarding the relationship between the 5:2 diet and circadian rhythm are still lacking. In addition, although studies in animals and humans suggest that the benefits of IF are mediated partly by the intestinal microbiota and its metabolites, a gut microbiota‐mediated mechanism awaits further investigation.^[^ ^110^, ^121^, ^172^ ^]^

### 2.3 Fasting‐Mimicking Diet

Implementing prolonged CR or complete fasting is often a challenge for humans. In addition, long periods of daily fasting (>15 h) may cause some adverse effects in certain pathological conditions, such as an increase in the mortality of patients with cancer and circulatory diseases.^[^ ^173^, ^174^ ^]^ To make fasting more acceptable to most people and achieve similar effects of fasting, FMD is mentioned.^[^ ^41^ ^]^ FMD is characterized by decreasing caloric intake for only 5 days each month. For a typical FMD protocol, the caloric amount is ≈1090 kcal (10% protein, 56% fat, and 34% carbohydrate) on the first day, and subsequently, 725 kcal is provided (9% protein, 44% fat, and 47% carbohydrate) for days 2 to 5 (Figure 1).^[^ ^41^ ^]^ This pattern is different from that seen with CR, which tends to reduce daily total energy intake and requires long‐term adherence.

A large number of studies indicate that FMD is strongly associated with immunological regeneration. FMD can promote lymphocyte circulation, improve immunological factors, enhance antitumor immunity and reduce autoimmunity (Figure 1).^[^ ^175^, ^176^, ^177^, ^178^, ^179^ ^]^ Lymphocytes play an important role in the tumor microenvironment by regulating antitumor immunity. Among them, CD8^+^ T lymphocytes are responsive to chronic infections and tumor antigens and act as a crucial mediator of the antitumor response.^[^ ^180^ ^]^ After three cycles of FMD, CD8^+^ T cells are rapidly increased in the bone marrow, together with a 2‐fold increase in common lymphoid progenitor cells.^[^ ^177^ ^]^ In addition, 4‐month cycles of FMD lead to increases in the number of red blood cells, the levels of hemoglobin, and the contents of some cytokine adjuvants, including interleukin 12 (IL‐12) and C‐C motif chemokine ligand 5.^[^ ^41^ ^]^ Furthermore, FMD cycles can reduce the mRNA levels of inflammatory factors, including Interferon γ, IL‐17, and tumor necrosis factor α (TNF‐*α*).^[^ ^178^ ^]^ It is noticeable that the immune system comprises numerous cell types and is very intricated, and the effect of FMD on immunological regeneration warrants future investigation.

**Figure 4:**
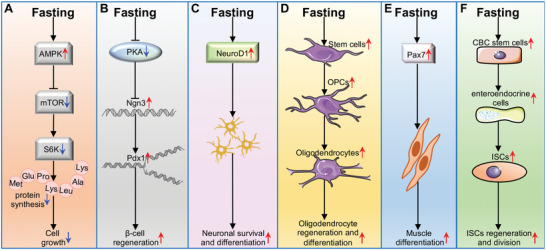
Fasting can affect the regeneration and differentiation of tissue and cells. A) FMD inhibits cell growth by regulating the AMPK‐mTOR signaling pathway; B) FMD modulates increased expression of Ngn3 which is an early developmental marker and leads to the regeneration of insulin‐producing *β* cells; C) FMD improves neuronal survival and differentiation neuronal survival and differentiation in brain tissue via inducing NeuroD1 expression; D) FMD stimulates regeneration and differentiation of oligodendrocytes and protects OPCs from apoptosis; E) FMD drives muscle differentiation via activating the expression of Pax‐7, which is a stem cell marker mainly expressed by muscle satellite stem cells; F) FMD promotes ISCs regeneration and division in part by increasing the numbers of crypt base columnar and enteroendocrine cells in intestinal tissue.

In addition to immunological regeneration, FMD may contribute to rejuvenating effects on stem cells and other systems, including brain, muscle, pancreas, and gut (**Figure** **4**). In general, fasting usually activates AMPK, the sensor of energy metabolism, and subsequently suppresses mTOR, resulting in reduced cell growth (Figure 4A).^[^ ^59^, ^151^, ^181^, ^182^ ^]^ Intriguingly, numerous studies have suggested a promotive role of FMD in stem cell differentiation and regeneration. FMD can reduce PKA and mTOR activity and then increase the expression of *β*‐cell reprogramming markers, including neurogenin 3 (Ngn3), and pancreatic and duodenal homeobox 1 (Pdx1), thereby driving generation of insulin‐producing *β* cells (Figure 4B).^[^ ^183^ ^]^ Interestingly, FMD is capable of increasing neuronal differentiation 1 (NeuroD1), a transcription factor critical for neuronal protection and differentiation, and thus improves hippocampal neurogenesis (Figure 4C).^[^ ^41^ ^]^ Meanwhile, FMD can enhance the differentiation of aged oligodendrocyte progenitor cells (OPCs),^[^ ^184^ ^]^ and also promote oligodendrocyte regeneration in experimental autoimmune encephalomyelitis (Figure 4D),^[^ ^178^, ^179^ ^]^ a model for MS, albeit the underlying mechanisms are currently unknown. In mouse skeletal muscle, FMD can restore the expression of paired box 7 (Pax‐7), a muscle stem cell marker, and thus increase quiescent satellite cell differentiation (Figure 4E).^[^ ^41^ ^]^ In addition, FMD can promote the development and differentiation of ISC and thus enhance gut regeneration by increasing certain protective microbial families, including *Lactobacillaceae* and *Bifidobacteriaceae* (Figure 4F),^[^ ^185^ ^]^ consistent with the emerging effect of FMD on gut microbiota composition.^[^ ^186^ ^]^ Despite these advances, the majority of current results concerning FMD and regeneration/differentiation are descriptive, and it is of great importance to elucidate the underlying mechanisms, which would throw insight into the physiological and pathological effects of FMD, as well as stem cell therapy.

In summary, these studies indicate that FMD cycles induce long‐lasting beneficial and rejuvenating effects on many tissues, including those of the immune and endocrine systems, in both mice and humans. Contrary to TRF and IF, current research efforts on FMD predominantly focus on pathological aspects, especially tumorigenesis. In this regard, the implication of FMD in physiological states, as well as in cellular metabolism and metabolic diseases, would be explored in the future.

## 3 Fasting in Pathology

In the past few decades, the physiology of fasting has been broadly investigated. In addition, an increasing body of evidence suggests that fasting is an effective nonpharmacological intervention for counteracting multiple diseases. Therefore, it is essential to explore the beneficial effects of fasting, as well as its implications, in human diseases and aging.

### 3.1 Metabolic Diseases and Their Complications

Obesity is a major driver of metabolic diseases, such as type 2 diabetes mellitus (T2DM), fatty liver diseases, and hyperglycemia. Overnutrition, fast food, and sedentary lifestyles are always connected to an increased risk of obesity.^[^ ^187^, ^188^ ^]^ Expectedly, increasing evidence strongly indicates that fasting has a protective effect on metabolic diseases and their complications by controlling body weight, enhancing organ function, and improving systemic metabolism.^[^ ^5^, ^183^, ^186^ ^]^ Extensive studies have been performed on the effects of fasting on body weight in obese mice.^[^ ^5^, ^189^ ^]^ Fasting suppresses body weight gain in diet‐induced obese mice.^[^ ^190^, ^191^ ^]^ Many human studies recapitulate the results from animal experiments.^[^ ^192^, ^193^, ^194^, ^195^ ^]^ For instance, a significant decrease in body weight has been reported for 8–10 h TRF protocols in pilot studies (Table 2).^[^ ^195^, ^196^, ^197^, ^198^ ^]^

Recent data indicate that fasting may prevent obesity by decreasing fat accumulation without causing energy imbalance.^[^ ^199^, ^200^ ^]^ In flies, fasting was shown to enhance lipolysis by maintaining a feedback loop between FOXO and Rlish.^[^ ^86^ ^]^ In mice, fasting can promote lipid mobilization and decrease fat deposition by inhibiting the expression of KLF15 (Figure 2).^[^ ^91^ ^]^ Moreover, fasting induces adipose tissue remodeling, including promoting WAT browning and angiogenesis.^[^ ^122^, ^154^, ^201^, ^202^ ^]^ Different types of adipose tissues have distinct phenotypes in response to fasting. In general, subcutaneous WAT (scWAT) releases fatty acids that provide energy for other organs and tissues during the fasting period. In contrast to scWAT, visceral WAT is resistant to the fasting‐reduced release of fatty acids and acts as an important safeguard to protect the organism against unwanted side effects of fasting.^[^ ^203^ ^]^ Although fasting is effective in weight loss and promoting lipolysis in high‐fat diet (HFD)‐induced obese mice,^[^ ^59^, ^204^ ^]^ it has no obvious effect on genetically obese/diabetic mice: an IF regimen of 1 day of fasting followed by 2 days of free feeding had no effect on body weight, hepatic lipid accumulation, or WAT beiging in ob/ob mice.^[^ ^205^ ^]^ 7 months of IF or 8 weeks of FMD also failed to reduce the body weight of db/db mice.^[^ ^127^, ^186^ ^]^ These results suggest that a single fasting regimen is not recommended as a weight control approach for inherited obesity and that a combination of fasting and exercise or pharmacological intervention may be favorable.

Studies in animals and clinics show that fasting is beneficial to metabolic diseases, particularly metabolic syndrome, T1DM/T2DM, and non‐alcoholic fatty liver disease (NAFLD) (**Table** **1**). The efficacy of fasting in improving metabolic syndrome has only been tested in TRF and IF. The results of these trials revealed that two fasting regimens are effective in reducing body weight.^[^ ^77^, ^110^, ^196^ ^]^ Studies in mice and humans with metabolic syndrome have suggested that TRF may have positive effects on blood pressure, cholesterol and glucose metabolism.^[^ ^77^, ^196^ ^]^ In contrast to TRF, IF does not alter these parameters and its benefits may mainly come from gut microbiota and its metabolites.^[^ ^110^ ^]^ Besides, the 3‐month TRF protocol failed to change blood biomarkers associated with metabolic syndrome for obese people, speculating that it may not be a suitable means of preventing metabolic syndrome.^[^ ^198^ ^]^ Fasting also exhibits protective effects on diabetes. Molecular studies of three fasting regimens on T2DM are predominately concentrated on the blood pressure circadian rhythm, *β* cell mass and function, as well as glucose and lipid metabolism in T2DM mice, including diet‐induced and inherited deficiency in the leptin receptor (i.e., db/db) mice.^[^ ^67^, ^121^, ^153^, ^183^, ^186^ ^]^ Notably, only FMD has been examined on mouse models of T1DM.^[^ ^183^, ^186^ ^]^ Clinically, TRF and FMD are associated with better blood pressure and glycemic control in individuals with diabetes.^[^ ^85^, ^103^, ^206^, ^207^ ^]^ Other metabolic parameters, such as triglycerides, cholesterol, and low‐density lipoprotein cholesterol (LDL‐C) would be similarly improved in most of these studies.^[^ ^103^, ^206^, ^207^ ^]^ The safety of fasting has been tested in two trials in humans. IF does not cause hypoglycemia for most individuals with T2DM on hypoglycaemic medication.^[^ ^208^ ^]^ Similar results were also achieved by FMD.^[^ ^209^ ^]^ Above all, because FMD is both effective and safe, it is a better treatment option for patients with T2DM. In addition, both TRF and IF can improve NAFLD. Studies in TRF mainly focus on animal models. A series of research revealed that TRF can decrease plasma triglycerides.^[^ ^210^, ^211^, ^212^ ^]^ But other NAFLD parameter, such as hepatic lipid, FFA, inflammation, are not consistent due to different experimental designs, differences in the size of study groups, and differences in the duration of the experiment. For instance, 2‐week of TRF protocol failed to decrease hepatic lipid accumulation but 4 weeks of TRF may contribute to lipid metabolism.^[^ ^211^, ^212^ ^]^ Interestingly, TRF reduced adiposity and normalized hepatic triglyceride for mice with context of childhood obesity, may contribute to improve NAFLD.^[^ ^210^ ^]^ Different from TRF, IF has been more practice in clinic trials. Most of the research suggests that IF is effective in reducing hepatic steatosis and fat mass, thereby ameliorating the pathogenesis of NAFLD.^[^ ^114^, ^213^, ^214^ ^]^ Mechanically, IF can activate Fgf2/PPAR*α* signal and elevate the expression of FAO‐related genes.^[^ ^150^, ^215^ ^]^ Based on these studies, we speculate that IF may act as a better non‐pharmacological dietary approach to use in clinical practice in alleviating NAFLD.

**Table 1:** Fasting regimens in health and diseases

Fasting regimens	Lifespan	Neuro‐degenerative diseases	Effects	Metabolic disorder	Effects	Cancer	Effects
TRF	Flies: 13–18%^[^ ^249^ ^]^ Mouse: 11%^[^ ^251^ ^]^	AD^[^ ^342^ ^]^	A*β* deposition↓ cognitive function↑	Metabolic syndrome^[^ ^77^, ^196^, ^198^ ^]^ T2DM^[^ ^67^, ^85^, ^103^, ^343^ ^]^ NAFLD^[^ ^210^, ^211^, ^212^, ^322^, ^344^ ^]^	Weight↓ body fat↓ circadian rhythm↑ inflammation↓	Breast cancer^[^ ^222^, ^345^ ^]^; lung cancer^[^ ^346^ ^]^	Diurnal gene expression rhythms↑ angiogenic factors↓
IF	Worm: 40.4–77.0%^[^ ^245^, ^246^ ^]^ Flies: 10%^[^ ^247^ ^]^ Mouse: 12.6–20.0%^[^ ^250^, ^252^ ^]^	AD^[^ ^165^, ^166^, ^300^, ^302^, ^347^ ^]^; HD^[^ ^348^ ^]^; MS^[^ ^120^, ^349^ ^]^	Cognitive function↑ neuronal differentiation↑ A*β* deposition↓ BDNF↑	Metabolic syndrome^[^ ^110^ ^]^ T2DM^[^ ^121^, ^153^, ^208^ ^]^ NAFLD^[^ ^114^, ^213^, ^214^, ^215^ ^]^	Steatosis↓ glycemic control↑ *β*‐cell mass↑ probiotic↑	ALL^[^ ^350^ ^]^; melanoma; colonic cancer^[^ ^227^ ^]^; breast cancer^[^ ^230^, ^351^ ^]^	LEPR signaling PP2A‐GSK3*β*‐MCL‐1 signal↑ serum glucose levels↓
FMD	Mouse:11–19%^[^ ^41^, ^193^ ^]^	PD^[^ ^305^ ^]^; MS^[^ ^178^, ^179^ ^]^	BDNF↑ inflammation↓ remyelination in axons↑	T1DM^[^ ^183^, ^186^ ^]^ T2DM^[^ ^183^, ^186^, ^206^, ^207^, ^352^ ^]^	*β*‐cell regeneration↑ ipolysis↑	Breast cancer;^[^ ^175^, ^233^, ^259^, ^324^ ^]^ melanoma;^[^ ^177^ ^]^ colorectal cancer^[^ ^176^ ^]^; lung cancer^[^ ^323^, ^334^ ^]^; prostate cancer^[^ ^353^ ^]^	IGF‐1; HO‐1↓ AKT‐mTOR↓ nutritional stress↑ autophagy↑

AD: Alzheimer's disease; AKT: AKT serine/threonine kinase; ALL: Acute lymphoblastic leukemia; BDNF: brain‐derived neurotrophic factor; FMD: fasting‐mimicking diet; GSK3β: glycogen synthase kinase 3β; HD: Huntington’s disease; HO‐1: hypoeretin‐1; IGF‐1: insulin‐like growth factor 1; IF: intermittent fasting; MCL‐1: LEPR: leptin‐receptor; MCL1 apoptosis regulator, BCL2 family member; MS: Multiple sclerosis; mTOR: mammalian target of rapamycin; NAFLD: Nonalcoholic fatty liver disease; PD: Parkinson's disease; PP2A: protein phosphatase 2 phosphatase activator; TRF: time‐restricted feeding; T1DM: type 1 diabetes mellitus; T2DM: type 2 diabetes mellitus.

**Figure 5:**
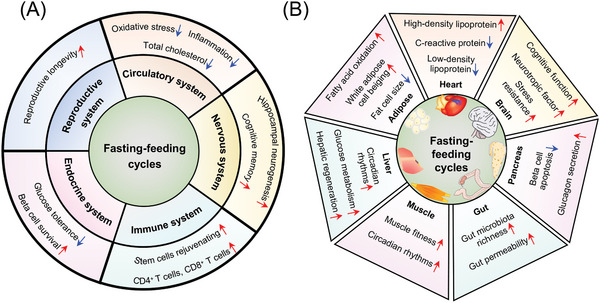
Pervasive benefits of fasting. A) Five systems are directly involved in the benefits of fasting. Fasting rescues mice from diabetes by decreasing glucose tolerance and increasing pancreatic *β* cell survival. The enhancement of reproductive longevity is a common characteristic of fasting physiology. Fasting reduces oxidative stress, inflammation, and total cholesterol in the circulatory system. HSC self‐renewal is also increased by rapid regeneration and rejuvenation of HSCs. Increased CD4+ and CD8+ T cells enhance anticancer immunity in the host. Fasting is also powerful for increasing cognitive ability and memory; B) More detailed benefits of fasting in several organs are outlined to elaborate its effects on systems as shown in (A), including but not limited to brain, heart, liver, muscle, adipose tissue, pancreas and gut. During fasting, levels of blood glucose and leptin are decreased, and the pancreas secretes glucagon. Fasting maintains the normal level of cellular autophagy during the aging process and improves cognitive function, stress resistance, and neurotrophic factors in the brain. The reduction in gut microbiota richness and diversity during aging is reversed by fasting.

In addition, obesity is associated with an increased risk of cardiovascular events. Numerous studies have demonstrated that fasting is effective for maintaining cardiac function and preventing cardiovascular disease (CVD). In flies and mice, TRF or IF can attenuate aging‐associated cardiac decline, improve systolic blood pressure, and maintain cardiac structure (**Figure** **5**B).^[^ ^216^, ^217^ ^]^ In humans, FMD can restore the levels of C‐reactive protein (CRP), a risk factor for CVD, and thus reduce the morbidity of CVD (Figure 5B, **Table** **2**).^[^ ^185^ ^]^ In line with these results, LDL‐C and high‐density lipoprotein cholesterol (HDL‐C), two important CVD biomarkers, were found to be significantly increased and decreased after 8 weeks or 12 months of IF, respectively (Table 2).^[^ ^110^, ^218^ ^]^ Although these studies show that fasting can improve these CVD‐related parameters, other studies about patients with diabetes could be inconsistent. For example, 5 weeks of TRF does not affect LDL‐C and HDL‐C in men with prediabetes.^[^ ^85^ ^]^ Another study found that 12 weeks of TRF reduced LDL‐C rather than HDL‐C in overweight adults with T2DM.^[^ ^103^ ^]^ Therefore, clinical protocols should be individualized, based on the patient's specific clinical indication.

**Table 2:** Clinical trials of fasting regimens

Fasting regimen	Combination	Participants	Duration	Benefits	BW change	Ref.
TRF	–	16 young cyclists	4 weeks	↓ fat mass ↓ free testosterone ↓ IGF‐1	−1.4 kg	[194]
TRF	–	22 college‐age men	4 weeks	↓ blood pressure ↑adiponectin, HDL‐C	–	[333]
TRF	–	24 healthy people	6 weeks	↑cardiorespiratory function ↓ glucose tolerance	–	[314]
TRF	–	10 overweight, older adults	4 weeks	↑walking speed ↑physical function	−2.6 kg	[197]
TRF	–	23 obese adults	12 weeks	↓systolic blood pressure	−2.6%	[195]
TRF	–	58 obese women	3 weeks	↓body fat ↓waist circumference	–	[319]
TRF	–	58 obese women	12 months	↓body fat	–	[320]
TRF	–	50 adults with overweight	3 months	↓systolic blood pressure	−2.1 ± 3.0 kg	[354]
TRF	–	9 men with prediabetes	5 weeks	↑insulin sensitivity ↓blood pressure ↓oxidative stress	−1.4 ± 1.3 kg	[85]
TRF	–	14 subjects with metabolic syndrome	4 weeks	↑tumor suppressor (eg. CALU, INTS6, KIT) ↓tumor promoter (eg. POLK, CAMP)	−3.3 kg	[355]
TRF	–	19 patients with metabolic syndrome	14 weeks	↓blood pressure ↓atherogenic lipids ↓LDL‐C	−3.30 ± 3.20 kg	[196]
TRF	–	120 overweight adults with T2D	12 weeks	↑*β*‐cell function ↓HbA1c level ↓insulin resistance ↓triglycerides, LDL‐C	−2.98 ± 0.43 kg	[103]
TRF	–	18 patients with polycystic ovary syndrome	5 weeks	↓hyperandrogenemia ↑insulin sensitivity ↓chronic inflammation	−1.3 kg	[356]
TRF	–	2413 women with breast cancer	1–4 years	↓HbA1c level ↓recurrence	−0.04%	[357]
IF	–	90 healthy adults	4 weeks/6 months	↓fat mass, LDL‐C ↓*β*‐hydroxybutyrate ↓inflammation	−3.5 ± 1.5 kg	[109]
IF	–	10 obese adults with moderate asthma	8 weeks	↓oxidative stress ↓inflammation	−8.5 ± 1.7	[328]
IF	–	26 obese adults	2 years	↓fasting glucose ↓total cholesterol, LDL‐C	−8.2 ± 0.9 kg	[312]
IF	–	28 obese males	1 month	↓IL‐6 ↓TNF‐*α*	–	[358]
IF	–	30 obese males	1 month	↑gut hormone levels (GLP‐1, PYY, CCK)	−2.3 ± 0.4 kg	[359]
FMD	–	100 healthy adults	3 months	↓body fat, IGF‐1 ↓blood pressure	−2.6 ± 2.5 kg	[329]
TRF	Exercise	12 healthy men	3 weeks	↑muscle performance	–	[313]
TRF	Exercise	20 healthy subjects	12 months	↑insulin sensitivity ↓body mass ↓inflammation	−2.56 ± 1.28 kg	[360]
TRF	Exercise	18 endurance trained males	9 weeks	↑biceps brachii ↑rectus femoris	–	[101]
TRF	Exercise	27 endurance trained males	4 weeks	↓fat mass ↓circulating lactate	–	[361]
TRF	Exercise	34 endurance trained males	8 weeks	↑adiponectin ↓fat mass, total leptin ↓testosterone, IGF‐1	−0.97% ± 1.58	[362]
TRF	L‐DOPA or/and DA	24 patients with PD	2 weeks	↓PD deterioration	–	[363]
TRF	Tomato juice	52 obese children with NAFLD	2 months	↓HOMA‐IR ↓LDL‐C, triglyceride ↑HDL‐C	−4 kg	[364]
IF	Probiotic	26 patients with prediabetes	12 weeks	↓HbA1c ↓leptin	−2 ± 1 kg	[365]
IF	Chemo‐therapy	13 patients with stage II/III HER2‐negative breast cancer	24 hrs	↓DNA damage	–	[325]
FMD	Metformin + chemo‐therapy	88 patients with LKB1‐inactivated lung adenocarcinoma	12 weeks	↑CD8^+^ T lymphocytes ↓glucose, insulin	–	[323]
FMD	Metformin	100 T2D patients	4 months	↑HDL‐C ↓blood pressure ↓triglyceride; LDL‐C	−5.11 kg	[206]
FMD	Metformin	100 T2D patients	1 year	↓HbA1c ↓medication dosage	–	[209]
FMD	Antitumor therapies	101 patients with different cancer types	8 months	↓glucose, insulin ↑CD8^+^ T lymphocytes ↑CD68^+^ macrophages	–	[37]
FMD	Chemo‐therapy	129 patients with stage II/III HER2‐negative breast cancer	1 month	↓tumor cells (90–100%) ↓CD45^+^ T lymphocytes ↓CD3^+^ T lymphocytes	–	[324]

BW, body weight; CAMP: cathelicidin antimicrobial peptide; CALU: calumenin; FMD, fasting‐mimicking diet; HbA1c, hemoglobin A1c; HDL‐C, high‐density lipoprotein cholesterol; HOMA‐IR, homeostasis model assessment of insulin resistance; IF, intermittent fasting; IGF‐1, insulin‐like growth factor 1; IL‐6, interleukin 6; INTS6: integrator complex subunit 6; KD, ketogenic diet; LDL‐C, low density lipoprotein cholesterol; PD, Parkinson's disease; POLK: DNA polymerase kappa; TNF‐*α*, tumor necrosis factor *α*; TRF, time‐restricted feeding.

### 3.2 Cancer

Cancer causes a tremendous burden to the health care system due to its high morbidity and substantial mortality. Metastasis, recurrence, and drug resistance are still major obstacles in the treatment of cancer.^[^ ^159^, ^219^ ^]^ In recent years, fasting has attracted considerable attention for the concept of “starving a tumor to death.” Although several early studies have shown that IF fails to inhibit tumor growth and improve survival in mouse models,^[^ ^220^, ^221^ ^]^ recent advances have demonstrated that fasting alone is effective for mitigating tumorigenesis and inhibiting tumor progression for certain types of cancer. For example, TRF reduced the lung metastasis of obesity‐driven breast cancer in mice by increasing insulin sensitivity and restoring circadian rhythms,^[^ ^222^ ^]^ and nightly fasting for more than 13 h significantly decreased the risk of breast cancer recurrence.^[^ ^223^ ^]^

**Figure 6:**
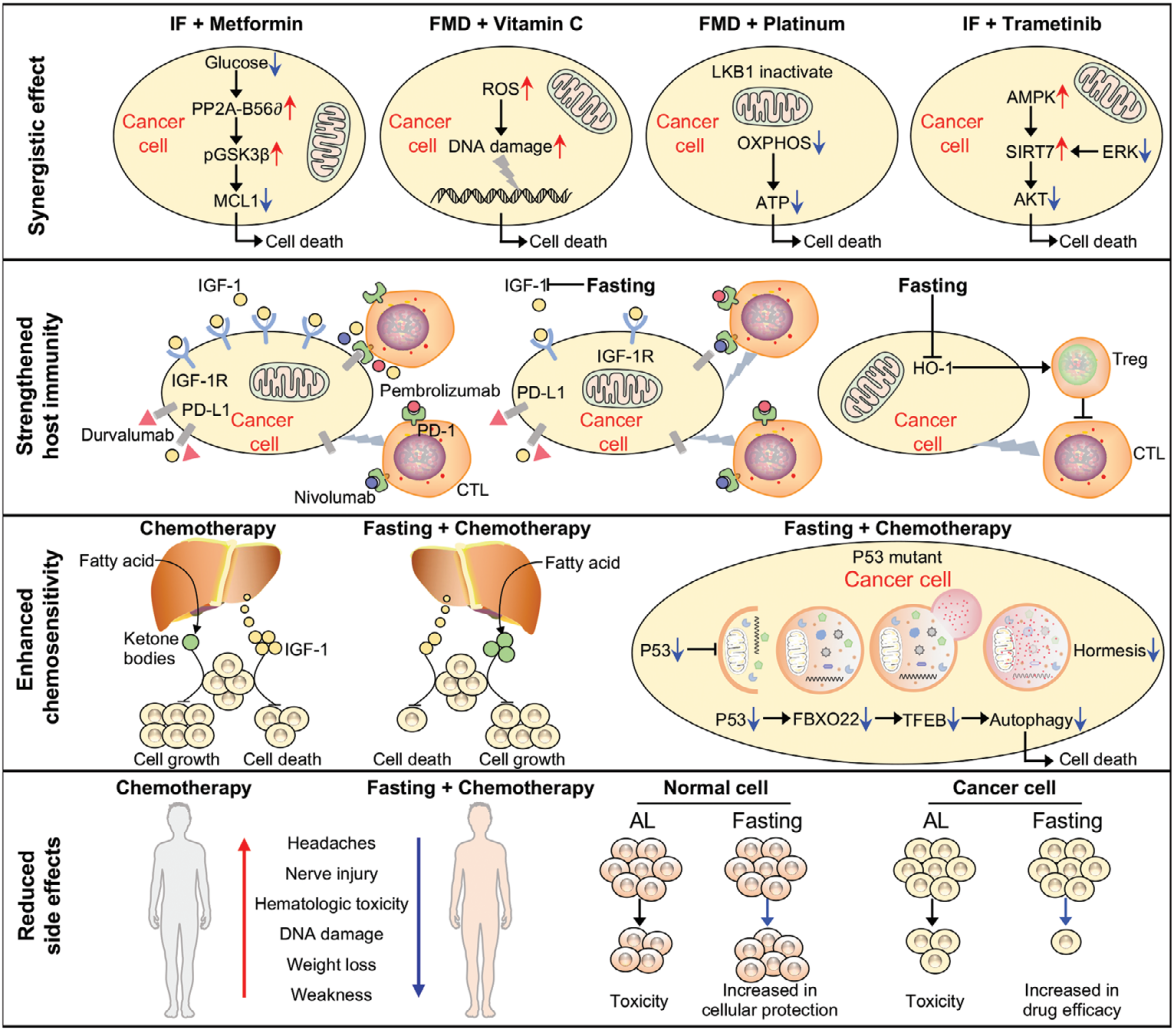
Fasting protects the body from the toxicity of chemotherapy while improving therapeutic efficacy. The positive effects of fasting on chemotherapy are in reflected four aspects: synergistic effects with pharmacological intervention, strengthened host immunity, enhanced sensitivity of tumor tissues or cells to chemotherapy, and reduced unwarranted side effects. Metformin, vitamin C, platinum, and trametinib have been shown to enhance the effectiveness of chemotherapy when they are administered in combination with fasting. Additionally, fasting results in a significant reduction in IGF‐1, thereby blocking the activation of PD‐1 and PD‐L1 signaling when combined with PD‐1/L1 inhibitors. In addition, during periods of fasting, hepatic metabolism is programmed to oxidize fatty acids and deliver fuel in the form of ketone bodies that progressively inhibit cancer cell growth. Fasting‐induced autophagy protects healthy cells from cancerous transformation and increases the apoptosis sensitivity of cancer cells. Furthermore, fasting alleviates certain side effects of chemotherapy, including headaches, hematologic toxicity, and weight loss. The figure, from Servier Medical Art (Creative Commons attribution license), was modified.

**Figure 7:**
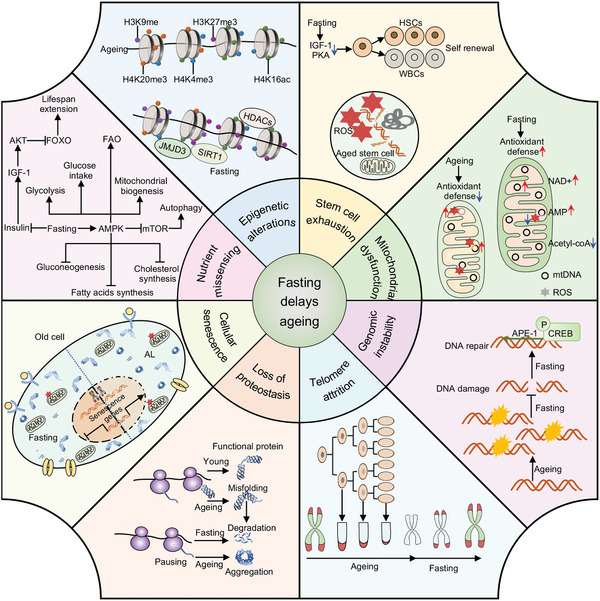
Fasting delays aging. Fasting retards aging by inhibiting a series of hallmarks of aging, including deregulated nutrient sensing, epigenetic alterations, genomic instability, telomere attrition, mitochondrial dysfunction, loss of proteostasis, stem cell exhaustion, and cellular senescence.

A major advantage of fasting has been reflected in the synergistic effect with pharmacological intervention on suppressing tumor metabolism, strengthening host immunity, and enhancing the sensitivity of cancer cells to chemotherapy (**Figure** **6**). First, the “Warburg effect,” a metabolic feature of tumor cells, is defined as an increased dependency of cancer cells on glycolysis even in the presence of oxygen.^[^ ^224^ ^]^ Suppression of aerobic glycolysis can help to enhance the effectiveness of chemotherapy. Both in vitro and in vivo evidence have indicated that fasting can inhibit glycolysis and promote cancer cell death following chemotherapy treatment.^[^ ^225^, ^226^ ^]^ Moreover, metformin plus fasting‐induced hypoglycemia impairs tumor metabolic plasticity.^[^ ^227^ ^]^ Inhibiting mTOR and blocking the phosphoInositide‐3 kinase (PI3K)/AKT/mTOR signaling pathway enhances chemosensitivity in many malignant tumors.^[^ ^228^, ^229^ ^]^ Fasting induces the expression of farnesyl‐diphosphate farnesyltransferase 1, thus repressing AKT/mTOR/hypoxia inducible factor 1 subunit alpha signaling and preventing tumor progression.^[^ ^225^ ^]^ In addition, FMD plus the MEK inhibitor trametinib can enhance the antitumor efficacy of doxorubicin by activating the glycogen synthase kinase 3 beta (GSK3*β*)/SIRT7 axis and inhibiting AKT activity.^[^ ^230^ ^]^ Second, a critical feature of cancer cells is their capability to escape the innate immune response and adaptive immune surveillance.^[^ ^231^, ^232^ ^]^ As mentioned above, FMD cycles can improve the regeneration and rejuvenation of the immune system by reversing the age‐dependent decline in the lymphoid‐to‐myeloid ratio and increasing the number of red blood cells and hemoglobin levels.^[^ ^41^ ^]^ Fasting also decreases the levels of insulin, leptin, and IGF‐1, and promotes development of CD8‐positive T lymphocyte precursors, which collectively enhance immunity.^[^ ^37^, ^177^ ^]^ Third, fasting is also able to strengthen the sensitivity of cancer cells to pharmacological therapy (**Figure** **7**). Preclinical models have shown that about 80–90% of tumor growth is inhibited with the combination of chemotherapy and fasting, compared with about 40–50% with chemotherapy alone.^[^ ^175^ ^]^ For triple‐negative breast cancer, FMD activates starvation escape pathways in cancer cells and reduces the surface markers of cancer stem cells, leading to increased recognition by chemotherapeutic agents.^[^ ^233^ ^]^ Many combinations have been marketed in animal models and clinical studies, including TRF plus programmed cell death 1 (PD‐1) blockade, FMD plus kinase inhibitors or estrogen, IF plus ERK inhibition, and so on.^[^ ^230^, ^233^, ^234^ ^]^ Additionally, fasting regimens are being evaluated in combination with antineoplastic drugs in clinical trials, such as sorafenib, vemurafenib, and gefitinib.^[^ ^235^ ^]^ These studies collectively suggest a valuable strategy to explore the potential of fasting regimens in combinatorial therapy.

Another particular facet of fasting regimens is their capacity to reduce the unwarranted side effect of chemotherapy, including protecting normal cells from the cytotoxicity of chemotherapy and reducing chemotherapy related toxicities (Figure 6). Chemotherapy is a mainstay of treatment for solid tumors. However, the adverse effects of drugs, such as cardiotoxicity, neurotoxicity, and hepatotoxicity, impair the efficacy of these treatments.^[^ ^236^, ^237^ ^]^ Fasting has been suggested to inhibit chemotherapy‐related toxicity while protecting normal cells. In a mouse model of neuroblastoma xenotransplantation, 48 h of starvation before etoposide treatment protected normal cells but not cancer cells from toxicity.^[^ ^238^ ^]^ Moreover, mice treated with fasting and etoposide had less stress or pain and decreased mortality rates.^[^ ^238^ ^]^ 3 days of fasting before treatment can prevent a series of side effects caused by the chemotherapeutic agent irinotecan in mice, such as weight loss, lower activity, and diarrhea, while maintaining the antitumor efficacy of irinotecan.^[^ ^239^, ^240^ ^]^ In patients with gynecological cancer, subjects who underwent modified short‐term fasting during half of the chemotherapy cycles demonstrated reduced chemotherapy‐induced toxicities, including stomatitis, headaches, and fatigue.^[^ ^241^, ^242^ ^]^ Bone marrow suppression is another severe side effect of chemotherapy, which usually leads to a decrease in HSCs and blood cells (especially lymphocytes). Long‐term (48 h) fasting or FMD in mice reduces DNA damage in bone marrow stem cells and promotes HSC self‐renewal, thereby restoring hematopoietic regeneration and reversing immunosenescence.^[^ ^41^, ^157^ ^]^

It has been well documented that fasting plus chemotherapy may be considered a clinical treatment for cancer patients. However, as Clifton remarked, the effects of IF on human cancer incidence and prognosis remain unknown because of a lack of high‐quality randomized clinical trials.^[^ ^108^ ^]^

### 3.3 Aging

Aging is a highly complex and multifactorial pathophysiological process that occurs gradually over time and causes structural and functional alterations within an organism. Moreover, aging is a major risk factor for neurodegenerative disorders, especially MS, AD, and PD.^[^ ^243^, ^244^ ^]^ The financial burden of age‐related health disorders will increase as the world population ages, and effective preventive and therapeutic approaches are urgently needed. Although aging is an inevitable process, numerous studies suggest that fasting can extend the maximum lifespan in diverse animal species and delay or retard aging by resisting several hallmarks of aging, including deregulated nutrient sensing, epigenetic alterations, genomic instability, telomere attrition, mitochondrial dysfunction, loss of proteostasis, stem cell exhaustion, and cellular senescence (Figure 7).

It has been shown that multiple fasting strategies can significantly extend lifespan in various animal models (Table 1). In worms, IF successfully extends lifespan by activating the GLH‐binding kinase 1/BZIP domain‐containing protein (AP‐1) and GTP‐binding protein Rheb homolog 1/Fork‐head domain‐containing protein (DAF‐16) axes. AP‐1 and DAF‐16 coordinately modulate transcriptional changes and play a role in longevity.^[^ ^245^, ^246^ ^]^ Similar to results in C. elegans, the positive effect of fasting on lifespan has also been observed in fruit flies.^[^ ^247^, ^248^, ^249^ ^]^ A 2‐day fed:5‐day fasted IF increased lipid content in 60‐day‐old flies, leading to lifespan extension.^[^ ^247^ ^]^ In mammals, fasting may prolong mean lifespan by 11–20% for adult mice,^[^ ^41^, ^250^, ^251^ ^]^ and this effect might largely depend on the mouse genotype and age of fasting initiation.^[^ ^252^ ^]^ FMD can prevent early death caused by obesity in mice fed an HFD and restore a normal lifespan.^[^ ^193^ ^]^ Fasting‐mediated lifespan extension is a complex phenomenon attributed to the pleiotropic effects of various systems, and the exact mechanisms are poorly understood. It has suggested that the longevity‐promoting effect of fasting might be associated with the upregulation of longevity gene Klotho,^[^ ^253^ ^]^ the modulation of key metabolic pathways including insulin/IGF‐1 signaling (IIS) and SIRTs,^[^ ^41^, ^253^, ^254^ ^]^ the regeneration of multi‐systems.^[^ ^41^, ^253^ ^]^ and the delay of life‐limiting neoplastic disorders.^[^ ^41^, ^250^ ^]^ Although these studies on animals suggest that fasting can lead to lifespan extension, this conclusion still needs to be verified by prospective cohort human studies. Thus far there is no evidence of fasting and lifespan in humans, however, the function of fasting in delaying aging is undeniable. For example, prolonged fasting in humans can increase the circulating levels of aging‐related metabolites, indicating a potential role in slowing human aging.^[^ ^255^ ^]^ Increasing evidence suggests that fasting delays or retards aging by attenuating several hallmarks of aging, such as deregulated nutrient sensing, genomic instability, and cellular senescence (Figure 7).^[^ ^256^, ^257^ ^]^

As mentioned above, fasting is able to regulate various nutrient‐sensitive signaling pathways, including the AMPK, mTOR, and IIS pathways.^[^ ^59^, ^60^, ^258^ ^]^ The activity of AMPK is reduced during aging.^[^ ^259^ ^]^ whereas fasting‐triggered AMPK activation can enhance cellular glucose uptake via glycolysis and mitochondrial biogenesis, promote FAO, and restrict fatty acid synthesis (Figure 7).^[^ ^59^, ^260^ ^]^ As a downstream effector, mTOR is suppressed by AMPK;^[^ ^261^ ^]^ therefore, AMPK inhibition leads to the recovery of mTOR activity, which may in turn promote aging.^[^ ^262^ ^]^ Fasting, including TRF and IF, can suppress mTOR by enhancing AMPK activity to avoid excess energy expenditure and thus inhibit autophagy and delay aging.^[^ ^59^, ^258^ ^]^ The IIS pathway is the most evolutionarily conserved aging‐controlling pathway.^[^ ^263^ ^]^ Fasting results in a significant reduction in serum insulin and IGF‐1 and therefore regulates hepatic glucose and lipid metabolism.^[^ ^60^, ^85^, ^264^ ^]^ In addition, various studies have shown that impairing the IIS pathway can activate FOXO‐dependent lifespan extension, while promoting its activity can accelerate senescence in C. elegans.^[^ ^265^, ^266^, ^267^, ^268^ ^]^ Consequently, fasting slows the procession of aging partially by restoring the metabolic network comprised of multiple nutrient‐sensitive signaling pathways.

Fasting also has a role in maintaining genomic stability, facilitating DNA repair, protecting telomeres from oxidative damage, and preserving telomere length, which are associated with genome instability, a hallmark of aging (Figure 7). In older adults with mild cognitive impairment, a 36‐month IF regimen has been shown to reduce the level of oxidative stress by increasing superoxide dismutase activity.^[^ ^269^, ^270^ ^]^ Both TRF and IF can reduce DNA damage, which is related to the upregulation of key DNA repair proteins such as centrosomal protein 164 and apurinic/apyrimidinic endonuclease‐1.^[^ ^271^, ^272^ ^]^ In addition, DNA damage alters telomeres, resulting in telomere shortening, cellular senescence, and death. Although the relationship between fasting and telomeres is poorly understood, a recent study indicated that fasting can increase telomere length by suppressing mTOR signaling in planarian stem cells.^[^ ^273^ ^]^ These results strongly suggest that fasting could maintain genome stability through modulating multiple processes, although its direct and indirect effects and mechanisms await further investigation.

Meanwhile, fasting could inhibit mitochondrial dysfunction and delay cell senescence (Figure 7). Mitochondrial dysfunction and oxidative stress are characteristic features of both senescence and aging and have been implicated in several diseases, such as ischemia–reperfusion (IR) injury.^[^ ^274^, ^275^ ^]^ Fasting significantly protects against acute liver and kidney injury, as well as renal fibrosis, in animal models of IR.^[^ ^74^, ^276^, ^277^, ^278^, ^279^ ^]^ Mechanistically, IF can increase the levels of NADH: ubiquinone oxidoreductase subunit B8, cytochrome c oxidase subunit I, and dynamin 1 like, indicating a role in preserving mitochondrial homeostasis. IF can also upregulate catalase and glutamate‐cysteine ligase modifier subunit but downregulate NADPH oxidase 4 and 8‐hydroxy‐2 deoxyguanosine, thereby reducing IR injury‐induced oxidative damage.^[^ ^277^ ^]^ Similarly, TRF also reduces the amount of ROS and inhibits oxidative stress in hepatic IR injury.^[^ ^74^ ^]^ In addition, fasting robustly suppresses Ca^2+^‐dependent mitochondrial permeability transition pore opening by inhibiting the expression of complexes I, IV, and V of the mitochondrial oxidative phosphorylation system, resulting in the alleviation of renal injury.^[^ ^278^ ^]^ Overall, these treatments in nonhuman biomedical models demonstrate a remarkable effect of fasting on preventing mitochondrial dysfunction and oxidative stress, which might contribute to healthspan and lifespan.

The modulating role of fasting on epigenetic modifications, especially histone modifications, may also contribute to its impact on aging (Figure 7). Age‐associated epigenetic marks are characterized by increased histone H4K16 acetylation, H4K20 trimethylation, and H3K4 trimethylation, as well as decreased H3K9 methylation and H3K27 trimethylation (H3K27‐me3).^[^ ^257^ ^]^ Fasting can modulate histone methylation and subsequent gene transcription. For instance, fasting can induce the nuclear translocation of the histone demethylase Jumonji D3 (JMJD3) by the FGF21/PKA signaling, and thus epigenetically activate global autophagy‐network and *β*‐oxidation‐promoting genes, including Atg7, Atgl, carnitine palmitoyltransferase 1A, Fgf21, acyl‐CoA dehydrogenase medium chain, and Tfeb, through H3K27‐me3 demethylation, eventually leading to hepatic autophagy, lipid degradation, and fatty acid *β*‐oxidation.^[^ ^280^, ^281^ ^]^ Similar to H3K27‐me3, H3K9‐me3 is often correlated with inactive chromatin and represses gene expression. It has shown that a 3‐month IF regimen is capable of modulating genome‐wide H3K9‐me3 in the mouse cerebellum, orchestrating a plethora of transcriptomic changes associated with IF‐triggered metabolic switching, albeit the corresponding demethylase(s) remain unknown.^[^ ^282^ ^]^ Of note, lysine‐specific demethylase 3A (KDM3A) appears to have a role in fasting‐induced hepatic autophagy, in which it can activate autophagy genes by decreasing H3K9‐me2 levels.^[^ ^283^ ^]^ It is of interest to determine if KDM3A might cooperate with certain histone tri‐demethylases to erase H3K9 methylation during fasting to ensure proper autophagy and maintain cellular metabolic homeostasis. SIRT1 is a NAD‐dependent class III histone deacetylase and can activate the expression of numerous key clock transcription factors and metabolic regulators, including Per2, Bmal1, Ppar*α*, and Lxr*α*, through deacetylation.^[^ ^281^, ^284^, ^285^, ^286^, ^287^ ^]^ Recent data suggest that fasting often increases the expression and activity of SIRT1, which might contribute to some fasting‐induced transcriptional changes and phenotypes. However, it has also indicated that SIRT1 overexpression had no role in intensifying the adaptations to IF.^[^ ^288^ ^]^ In this regard, the contribution of SIRT1 in fasting‐induced epigenetic modifications and effects is controversial and awaits further investigation.

In addition, proteostasis may participate in the process of fasting‐modulated aging (Figure 7). Many studies have demonstrated that proteostasis is altered with aging, including the accumulation of unfolded, misfolded, or aggregated proteins.^[^ ^289^, ^290^, ^291^ ^]^ It is well known that cellular proteome stability is affected by AMPK, mTOR, IIS, and NAD‐dependent deacetylases.^[^ ^292^ ^]^ which could be modulated by fasting. Correspondingly, IF can suppress multiple elements in proteolytic pathways, including proteases, lysosomes, and autophagy and ubiquitin pathway components,^[^ ^153^, ^246^, ^293^ ^]^ and fasting can lead to a dramatic change in the liver proteome.^[^ ^294^ ^]^ Nevertheless, relatively little research is available on the correlation between fasting and proteostasis, and the underlying signaling pathways and molecules are poorly understood.

### 3.4 Neurodegenerative Diseases

Aging is a high‐risk factor for neurodegenerative diseases. The most common neurodegenerative diseases, AD and PD, are predominantly observed in elderly individuals, and the risk of these diseases increases with age.^[^ ^244^ ^]^ MS is also a chronic inflammatory and neurodegenerative disease in conjunction with aging that affects the central nervous system.^[^ ^295^, ^296^ ^]^ Aberrant neuronal network activity and neuronal loss are two common pathological features of neurodegenerative diseases,^[^ ^243^, ^244^, ^297^ ^]^ and inflammation and the gut microbiome also play a key role in the progression of neurodegenerative diseases.^[^ ^244^, ^298^ ^]^ Preventing these dysregulations thus represents a promising therapeutic avenue to combat neurodegenerative diseases. Indeed, recent studies both in animals and humans have shown that fasting can promote neurogenesis, lower chronic inflammation, and reshape the gut microflora, thereby alleviating neurodegenerative, including AD, PD, and MS.^[^ ^41^, ^179^, ^299^, ^300^, ^301^ ^]^

Fasting may prevent and alleviate AD mainly by regulating neuronal activity and neurogenesis (Table 1). Adult neurogenesis is considered to play an essential role in learning and memory, and impaired neurogenesis leads to cognitive deficits associated with AD.^[^ ^244^ ^]^ Fasting regimens, especially IF, show an outstanding ability to protect against neuronal loss and promote neurogenesis in the hippocampus (Figures 1 and 5). The neuroprotective effect of IF is mediated by multiple mechanisms. IF can upregulate the expression of BDNF and Klotho (an antiaging protein) and then promote the proliferation and differentiation of neuronal stem cells and hippocampal neurogenesis (Figure 5A).^[^ ^253^, ^299^ ^]^ IF also enhances GSK3*β* activity, thereby improving hippocampal neuronal differentiation and maturation.^[^ ^302^ ^]^ Furthermore, IF‐induced SIRT3 has the ability to improve hippocampal neuronal network adaptability.^[^ ^300^ ^]^ Additionally, fasting has been shown to delay cognitive impairments, improve hippocampus‐dependent memory, and slow the process of AD, although the underlying mechanisms remain unclear.^[^ ^253^, ^300^, ^303^, ^304^ ^]^

As mentioned above, fasting can lower chronic inflammation, which is beneficial for alleviating PD and MS. Fasting robustly decreases the levels of inflammatory markers (e.g., IL‐1*β*, C‐C motif chemokine ligand 2, and CRP) in a variety of mouse models (Figure 5A).^[^ ^276^, ^286^, ^305^ ^]^ Additionally, systemic inflammation, measured by IL‐6, TNF‐*α*, and IL‐1*α*, appears to be related to the phenomenon of low‐grade inflammation in healthy elderly individuals, known as inflamm‐aging.^[^ ^306^ ^]^ Interestingly, the effect of fasting on TNF‐*α* appears to be complex and may depend on the regimens of fasting. For instance, the level of TNF‐*α* was shown to decrease significantly only after fasting every other day, but this was not obvious after fasting for 2–4 days.^[^ ^276^ ^]^ Similar unchanged levels of TNF‐*α* were also observed in healthy lean men after short‐term (4 weeks) TRF treatment.^[^ ^307^ ^]^ These results indicate that the relationship between TNF‐*α* and fasting would be clarified in the future, emphasizing the type, onset, and duration of fasting. More recently, it has been shown that IF is able to suppress inflammation by blunting CD4^+^ T helper cell responsiveness and inhibiting Th1 and Th17 immune responses.^[^ ^308^ ^]^ FMD can reduce the number of T_H_1 and T_H_17 effector cells and the production of proinflammatory cytokines, while IF inhibits pathogenic cytokines (IL17, TNF‐*γ*, and TNF‐*α*) and thus decreases autoimmune responses.^[^ ^178^, ^179^, ^308^, ^309^ ^]^ The differentiation ability of OPCs has been shown to be improved by fasting, which subsequently promotes remyelination and reverses the disease course of MS (Figure 4A).^[^ ^184^ ^]^ Three cycles of FMD have been shown to inhibit neuroinflammation in PD mice by decreasing the number of glial cells, as well as the release of TNF‐*α* and IL‐1*β*.^[^ ^305^ ^]^ These findings indicate that the potent anti‐inflammatory effect of fasting may prevent the initiation and progression of neurodegenerative diseases.

In addition, fasting may suppress PD and MS by reshaping the gut microflora (Figure 5B). An increasing number of studies have been conducted to link gut microflora to neurological disorders.^[^ ^310^ ^]^ For example, the gut microflora of PD patients has lower levels of *Prevotellaceae* and *Lactobacillaceae* than those of healthy subjects. IF and FMD regimens increase the abundance of these strains and reduce the number of *Actinobacteria* and *Tenericutes*.^[^ ^120^, ^122^, ^185^ ^]^ FMD also modulates shifts in gut microbiota composition, including a higher abundance of *Firmicutes*, *Tenericutes*, and *Opisthokonta* and a lower abundance of *Proteobacteria*.^[^ ^305^ ^]^ Therefore, fasting can delay the course of PD, at least partly through altering gut microbial populations, although its precise role and mechanism underlying the pathology of PD, as well as other diseases, are far less clear. In mouse models of MS, IF and FMD increase the relative richness of the beneficial intestinal flora, especially *Lactobacillus*, which produces SCFAs associated with inflammation, thereby preventing MS.^[^ ^121^, ^185^ ^]^ Given the increasing significance of the gut microbiota, it is of great interest to its role and mechanism in fasting‐mediated effects on the pathogenesis of neurodegenerative diseases.

## 4 Clinical Studies

Numerous benefits of fasting have been demonstrated by substantial animal experimental results. However, its feasibility and safety must be evaluated by clinical studies. Currently, most clinical studies have focused on (1) the pathological effect of fasting on obesity and obesity‐associated metabolic disease; (2) fasting as an adjunct to cancer therapy; and (3) the safety of fasting. The effects of the three fasting regimens on clinical outcome measures are reported in Table 2.

Fasting is a valuable strategy for obese individuals, and the majority of clinical studies suggest that fasting can lead to weight loss. Three fasting regimens were effective for weight loss in individuals with obesity and overweight, inducing weight loss of about 3–8% from baseline.^[^ ^192^, ^198^, ^311^ ^]^ Both ADF and CR in adults with obesity have the same effect on weight loss, but ADF is more effective for improving fat mass and lean mass (**Table** **3**).^[^ ^312^ ^]^ Fasting also facilitated reductions in weight in individuals of normal weight, resulting in ≈0.9 kg of weight loss per week.^[^ ^109^ ^]^ but did not cause weight loss in lean healthy people.^[^ ^313^, ^314^, ^315^ ^]^ Of note, a person's sex or menopausal status does not appear to affect the effectiveness of fasting in reducing weight.^[^ ^316^ ^]^ The efficacy of fasting in reducing weight is also reflected in other populations, such as shift workers or obese individuals with sedentary lifestyles.^[^ ^197^, ^317^, ^318^ ^]^ In contrast, fasting fails to cause weight loss in obese subjects with low income.^[^ ^319^, ^320^ ^]^ Interestingly, both short‐term (81‐day) and long‐term (12‐month) TRF in low‐income women with obesity could result in a decrease in waist circumference, albeit no obvious effect on weight loss was observed, suggesting TRF as an alternative strategy for combating obesity socially vulnerable population.^[^ ^319^, ^320^ ^]^

**Table 3:** Combinated dietary strategies in human health and disease

Fasting regimen	Compared	Participants	Duration	Advantage	Disadvantage	Equivalence	Ref.
TRF	Orthodox fasting	37 overweight adults	7 weeks	–	Lipid lowering effects	Glycaemic control, inflammation	[366]
eTRF	mTRF	90 healthy volunteers without obesity	5 weeks	Insulin sensitivity; body mass; inflammation	–	–	[367]
IF	CR	65 adults with metabolic syndrome	12 weeks	–	–	Weight loss, blood pressure, triglyceride, LDL	[368]
IF	CR	88 obesity women	8 weeks	Weight loss	–	cognitive performance	[369]
IF	CR	43 adults with central obesity	4 weeks	Recognition memory	–	Cognitive improvement	[370]
IF	CR	76 obesity women	8 weeks	Lipid storage, weight loss	–	Lipid metabolism in muscle	[371]
IF	CR	62 women with a history of gestational diabetes	12 months	–	–	Weight loss	[372]
IF	CR	43 adults with central obesity	4 weeks	Fasting plasma non‐esterified fatty acid	Fasting plasma glucose	Weight loss, inflammatory markers, lipids,	[373]
IF	CR	24 healthy individuals with lean	3 weeks	Fat mass	–	Body mass	[315]
IF	Paleolithic diet/Mediterranean diets	250 overweight healthy adults	12 months	Weight loss	Glycated hemoglobin	Systolic blood pressure, body fat	[374]
IF+ exercise	CR+ exercise	34 participants	12 weeks	–	Muscle surface area	Body mass	[375]
FMD	CR	60 obesity women	2 months	Muscle mass, fat mass	Basal metabolic rate	Weight loss, insulin sensitivity	[376]

CR, calorie restriction; eTRF, early TRF; FMD, fasting‐mimicking diet; IF, intermittent fasting; LDL, low density lipoprotein; mTRF, mid‐day TRF; TRF, time‐restricted feeding.

IF is an effective therapy for patients with T2DM or NAFLD (Table 2), achieving improved glycemic control (Table 2). FMD is also feasible and effective to reduce body weight and improve metabolic homeostasis in T2DM patients.^[^ ^206^, ^207^ ^]^ Of note, for diabetes patients, it is essential to evaluate the risk of hypoglycemia before starting fasting regimens.^[^ ^321^ ^]^ For NAFLD patients, ADF is able to significantly reduce weight and improve dyslipidaemia.^[^ ^213^ ^]^ Similarly, the 5:2 diet is effective for weight reduction and improvements in hepatic metabolism and function, representing a more efficacious means of treating obesity‐induced NAFLD than general lifestyle advice.^[^ ^114^, ^214^ ^]^ Notably, the combination of TRF and a low‐sugar diet can markedly attenuate adiposity and improve liver dysregulation, lipid metabolism, and inflammation.^[^ ^322^ ^]^ In addition, fasting can contribute to preventing metabolic syndrome. For instance, TRF as a dietary intervention not only strengthens cardiometabolic health for patients with metabolic syndrome but also decreases blood pressure, total cholesterol, and LDL‐C in these patients (Table 2).^[^ ^196^ ^]^ However, it has also been shown that 4‐ or 6‐h TRF accompanied by reduced energy intake cannot affect blood pressure, LDL‐C, HDL‐C triglycerides, and inflammatory markers (e.g., TNF*α* and IL‐6) for patients with obesity.^[^ ^102^ ^]^ These inconsistent results may be due to the different characteristics of the study subjects or duration of fasting.

As mentioned above, fasting‐based therapies for tumors have achieved encouraging success in animal models.^[^ ^175^, ^176^, ^177^ ^]^ Some key findings from animal studies have been confirmed by clinical trials, such as breast cancer and lung cancer.^[^ ^223^, ^323^ ^]^ For patients with early‐stage invasive breast cancer, TRF can reduce the risk of recurrence by improving glycemic control when patients underwent nightly fasting for more than 13 h.^[^ ^223^ ^]^ For patients with HER2‐negative stage II/III breast cancer, as well as early‐stage invasive breast cancer, FMD or IF plus chemotherapy was shown to effectively inhibit tumor progression while attenuating chemotherapy‐induced DNA damage in T‐lymphocytes and peripheral blood mononuclear cells (Table 2).^[^ ^324^, ^325^ ^]^ The combination of FMD and metformin can enhance the efficacy of platinum‐pemetrexed chemotherapy in advanced LKB1‐inactivated lung adenocarcinoma, thereby prolonging the survival of patients.^[^ ^323^ ^]^ Furthermore, FMD in combination with standard antitumor therapies is safe and effective by reshaping metabolism and enhancing antitumor immunity for multiple cancer patients (Figure 6).^[^ ^37^ ^]^ However, current clinical data on fasting are quite limited, and future research is needed to validate and expand the feasibility and generalizability of fasting as a nonpharmacologic strategy for preventing cancer progression and recurrence.

In addition to metabolic disorders and cancer, the effect of fasting on other diseases, such as asthma, is starting to emerge. Asthma is an increasingly common disorder responsible for considerable morbidity and mortality, and its prevalence and incidence are positively associated with obesity.^[^ ^326^ ^]^ Research has indicated that fasting is capable of improving asthma remission by modulating glucose metabolism (Table 2).^[^ ^327^, ^328^ ^]^ For example, an IF regimen lasting 8 weeks for overweight asthma patients improved clinical pathological manifestations, including reduced levels of serum cholesterol and triglycerides, decreased oxidative stress, and increased levels of antioxidant uric acids.^[^ ^328^ ^]^

The safety of fasting continues to attract attention, in which clinical evaluation is particularly important. Several short‐term clinical studies have shown that fasting is associated with significant metabolic benefits, rather than few and mild adverse effects. In young, healthy, nonobese volunteers, several metabolic markers, such as organic acids, coenzymes, antioxidants, purines, and pyrimidines, were upregulated during 58 h of fasting.^[^ ^255^ ^]^ The systemic accumulation of metabolites can activate multiple metabolic pathways, including antioxidative defense, mitochondrial activity, purine and pyrimidine anabolism, and the pentose phosphate pathway.^[^ ^255^ ^]^ In a randomized controlled clinical trial, Wei et al. tested the effects of FMD on markers/risk factors associated with aging, diabetes, cancer, and CVD from 2013 to 2015 (Table 2).^[^ ^329^ ^]^ Cycles of FMD can reduce cardiometabolic risk factors, including body mass index, blood pressure, fasting glucose, IGF‐1, triglycerides, CRP, and body weight (Figure 6b, Table 2). Importantly, there was no serious adverse effect on subjects after three cycles. Similarly, Stekovic et al. evaluated the safety, outcomes, and molecular changes of fasting in a healthy population in a clinical trial.^[^ ^109^ ^]^ They found that IF reduced the inflammation marker intercellular adhesion molecule 1 and the circulating levels of triiodothyronine in healthy, middle‐aged humans. In particular, the IF protocol improved cardiovascular markers and reduced fat mass. Likewise, FMD cycles in combination with standard antineoplastic treatments in cancer patients were safe and feasible.^[^ ^330^ ^]^ These clinical trials have convincingly established that different types of fasting regimens appear to be safe. However, future research is needed to investigate the long‐term efficacy and safety of fasting.

## 5 Conclusions and Future Perspectives

Based on the existing evidence from animal and human studies described in this review, there is great potential for fasting regimens that incorporate TRF, IF, and FMD for reducing the risk of numerous diseases, particularly metabolic disorders, cancer, neurodegenerative diseases, as well as delaying the adverse health changes associated with aging. Animal studies have documented robust and sustained effects of fasting on health indicators, including increased insulin sensitivity, decreased blood pressure, reduced body fat, and improved glucose homeostasis and lipid metabolism.^[^ ^40^, ^331^, ^332^ ^]^ For healthy people, fasting improves systemic metabolic indices and enhances the function of multiple organs, thereby creating a virtuous cycle and retarding aging.^[^ ^109^, ^329^, ^333^ ^]^ Additionally, fasting can attenuate the disturbance of glucose and lipid metabolism, promote the regeneration of stem cells or organs, and inhibit disease progression in obese individuals and patients with metabolic syndrome.^[^ ^196^, ^198^ ^]^ Furthermore, upon coadministration with chemotherapeutic agents, fasting can decrease the associated side effects while enhancing chemotherapeutic efficacy.^[^ ^175^, ^324^ ^]^ Mechanistically, research in TRF has demonstrated that fasting‐related benefits are mainly achieved by entraining the peripheral circadian clock, including the liver, muscle, adipose tissue, and gut microbiome.^[^ ^35^, ^51^, ^80^, ^126^ ^]^ Despite the effect of IF on tissue/organ regeneration has been reported, more studies have focused on the role of the intestinal flora and gut‐mediated inter‐organs communications. Like IF, FMD can also promote the regeneration and differentiation of multiple tissues and cells, and strengthen antitumor immunity.^[^ ^41^, ^178^ ^]^ In addition, there is no denying that all three regimens can affect gut microbiome, autophagy, and mitochondrial function.^[^ ^37^, ^70^, ^122^, ^153^, ^186^, ^249^, ^300^, ^334^ ^]^

Although the benefits of fasting have been well documented, there are still many areas of fasting that merit further study. Fasting protects the organism by eliciting alterations in energy metabolism while enduring extended periods of food deprivation. In fact, convincing evidence indicates that fasting‐feeding cycles need to be consistent with cycles of sleep and wakefulness and the benefit of fasting‐feeding cycles may also result from refeeding.^[^ ^70^, ^199^, ^200^, ^335^ ^]^ However, there are few studies on the efficacy and mechanisms of refeeding in humans. The exploration of the molecular mechanism(s) of refeeding may enrich the development of fasting‐feeding cycles and provide new insights into fasting‐based therapeutics. Moreover, although the role of fasting in both health and diseases is well described in both animal models and human subjects, the exact metabolic, immune, circadian mechanisms by which fasting can lead to physiological changes and resistance to pathological states are incompletely understood. In addition, TRF can improve cellular metabolism irrespective of when the fasting window occurs (duration of fasting may be from 12 to 20 h), but there have been very few studies comparing the benefits of the duration of fasting or the time to start fasting daily. The effects of fasting on plasma inflammatory and metabolic markers are less clear, due to the variation in the duration of fasting from several hours to several days. For instance, some studies demonstrated a decrease in LDL‐C by fasting,^[^ ^109^, ^110^, ^196^ ^]^ while others showed no significant effect.^[^ ^85^, ^329^, ^336^ ^]^ It is also of interest to note that fasting often leads to weight loss, thus it is important for future studies to clarify the potential contribution of weight reduction on fasting‐mediated metabolic improvements.

The safety of fasting regimens for clinical application is essential. The vast majority of studies suggest that fasting regimens can result in some weight loss, despite their tremendous benefits. While weight reduction may have a significant effect on overweight populations, it would be detrimental for those patients with a body weight below normal. Fasting periods lasting longer than 24 h, particularly those lasting 3 or more days, could have a few side effects. Of note, despite a few clinical studies showing that FMD cycles for several months are safe and feasible,^[^ ^329^, ^330^ ^]^ it remains uncertain whether long‐term fasting is advisable. Thus, randomized controlled trials and qualitative studies running for multiple years will offer important insights into the benefits of these regimens in patients with diseases, such as T2DM, NAFLD, polycystic ovary syndrome, and thyroid disorders. In addition, multiple randomized trials have shown that CR and fasting have an equivalent benefit for weight loss, inflammation, and cognitive performance. More controlled clinical research is needed to test whether fasting regimens can complement or replace energy restriction and, if so, whether they can facilitate long‐term metabolic improvements and avoid side effects. Last but not least, more research is needed to evaluate the consequences of fasting on psychological parameters, such as mood and anorexic response, as well as the potential effects on offspring resulting from epigenetic memory.^[^ ^337^, ^338^, ^339^ ^]^

Fasting alone may not be possible to maximize the benefits, and a combined application, such as a combination of two or more fasting regimens and fasting plus other dietary strategies or lifestyle including endurance exercise, may provide promise for the development of disease treatments and health management. IF combined with CR can reduce the risk of coronary heart disease in obese women.^[^ ^340^ ^]^ As a result, more benefits of fasting plus CR should be further explored, such as delaying aging, preventing diabetes, and resisting obesity. A better understanding of the molecular mechanisms underlying fasting plus CR and refeeding is necessary. Furthermore, some novel combinations of fasting are worth considering. For example, given the positive effect of FMD and TRF on antitumor immunity and survival, respectively,^[^ ^175^, ^176^, ^222^ ^]^ it is of interest to investigate the potential of the combination of these interventions for the treatment of certain human cancers. Fasting plus endurance exercise is an area of intense current research. The combination of IF and high‐intensity interval training (HIIT) is likely to result in better body weight control compared to IF alone.^[^ ^204^, ^329^, ^341^ ^]^ Other metabolic indicators, including blood glucose, serum insulin level, homeostasis model assessment of insulin resistance (HOMA‐IR), HDL‐C, and LDL‐C, were improved with HIIT compared to IF alone.^[^ ^204^ ^]^ Given the feasibility and effectiveness of both fasting and endurance exercise, their combination is highly anticipated to become a rising star for preventing and treating multiple human diseases.

The clinical feasibility of fasting regimens is a great challenge. For many years, the efficacy and safety of fasting have been mainly limited to specific dietary patterns (i.e., from 5 p.m. to 10 a.m.), the duration of the regimen (i.e., lasting 12 weeks), and the clear nutritional composition (i.e., 50% fat, 30% carbohydrate and 20% protein). However, these interventions are not only burdensome but also difficult to achieve for many. Therefore, an important clinical and scientific question is whether adopting regular fasting is feasible and sustainable for most people. In addition, fasting plus chemotherapy is more effective for cancer treatment in mice. However, to date, there have been few studies on other pharmacological treatments and clinical trials. We look forward to the potential implications of fasting and drug therapy (not limited to cancer treatment) in clinical and precision medicine.

There have been great advances in our understanding of fasting in both physiology and pathology in recent years, but many conclusions are still novel or somewhat inconsistent. Further elucidation of the roles, mechanisms, and outcomes of fasting, with the benefits from progress of new technologies such as single‐cell multi‐omic approaches, together with the optimization of fasting regimens and their combinations with other therapeutic strategies, are expected to provide a novel paradigm for preventing and treating human diseases.

## Conflict of Interest

The authors declare no conflict of interest.
